# The ovine hepatic mitochondrial proteome: Understanding seasonal weight loss tolerance in two distinct breeds

**DOI:** 10.1371/journal.pone.0212580

**Published:** 2019-02-20

**Authors:** Blake A. Miller, Aspinas Chapwanya, Tanya Kilminster, Tim Scanlon, John Milton, Hugo Osório, Chris Oldham, Johan Greeff, Don R. Bergfelt, Alexandre M. O. Campos, André M. Almeida

**Affiliations:** 1 Department of Clinical Sciences, Ross University School of Veterinary Medicine, St. Kitts and Nevis; 2 Department of Agriculture and Food Western Australia, Perth, WA, Australia; 3 University of Western Australia, Perth, WA, Australia; 4 Instituto de Investigação e Inovação em Saúde, University of Porto, Porto, Portugal; 5 Institute of Molecular Pathology and Immunology at the University of Porto, Porto, Portugal; 6 Department of Pathology, Faculty of Medicine, University of Porto, Porto, Portugal; 7 Interdisciplinary Centre of Marine and Environmental Research, University of Porto, Matosinhos, Portugal; 8 Linking Landscape, Environmental, Agriculture and Food, Instituto Superior de Agronomia, University of Lisbon, Lisbon, Portugal; University of Balochistan, PAKISTAN

## Abstract

Seasonal weight loss (SWL) is a primary constraint for farmers in the Mediterranean and tropics. One cost-effective solution to SWL is utilizing breeds like the Damara sheep that have adapted to deal with nutritional stress. Previous studies concluded that one of the adaptation mechanisms of SWL is a specialized fatty acid metabolism. Accordingly, hepatic-mitochondrial proteomes were compared across two different breeds (24 sheep total, Merino, n = 12 and Damara, n = 12) and two different diets (restricted vs unrestricted diet, 6 per breed, per diet, 24 total). Mitochondrial-proteins were isolated and relatively quantified using Blue native PAGE / 2D-electrophoresis and then analyzed via mass spectrometry. The tool ReviGO summarized the proteomes’ gene-ontology terms. A total of 50 proteins were identified with 7 changing significantly in abundance (ANOVA p-value<0.05). Specific abundance patterns of corticosteroid and inflammatory response-associated proteins such as annexin and glutamate dehydrogenase suggests that the Damara has an unusual inflammation response when subjected to SWL in addition to its unique metabolism. All significant proteins warrant further study; Annexin in particular shows promise as a potentially useful biomarker.

## Introduction

Tropical and Mediterranean climates are well known for their wet and dry seasons [1]. Dry seasons result in poor quality pasture for grazing animals, which, consequently, results in seasonal weight loss (SWL). SWL represents major annual setback to animal production in these regions and is characteristic of production in Western Australia [1], Africa [2], particularly West Africa [3], the Canary Islands and more[4,5]. In such climates, grazing animals can lose up to 30% of their live weight during the dry season with severe consequences to farmer’s income. For instance in previous studies, we have estimated such losses on roughly 25–30% on both live weight and income [1] and in the case of dairy animals, milk production outputs may decrease to over 80% milk produced per animal [4]. Such limitations have necessarily strong implications on the livelihood and also nutritional status of local populations, very dependent on crops but also on domestic animals, particularly small species, for the supply of protein, as we have demonstrated in Guinea-Bissau [3]. Farmers who keep livestock in such areas must contend with this annual obstacle to maintain production.

Farmers in drought prone regions often make use of commercial feed supplements to meet the nutritional requirements of grazing animal, which are both expensive and difficult to implement [4,5]. They are unwieldly as long-term solutions as a result [6]. A more cost-effective approach would be to use animals that have an innate ability to tolerate nutritional stress due to pasture scarcity [6,7]. Many breeds currently kept in such regions are of European origin and are poorly adapted to food scarcity. While these breeds are often highly productive, they suffer under pasture scarcity and the production costs of their poor adaptation to arid conditions should be carefully considered. There is a need to better understand the nature of metabolic adaptations to nutritional stress and it how effects full systems physiology. This study aims to establish those relations by comparing and contrasting a breed poorly adapted to SWL conditions against a breed that is much better adapted. We propose that this groundwork could eventually lead to a selection index that can be further refined into a list of biomarkers useful for selection of stock that is more tolerant to SWL and SWL-like conditions.

This study utilizes the Damara, a breed of fat-tailed sheep that has evolved in the fringes of the Kalahari Desert in SW Africa (Namibia and South Africa). It has since been translocated to other regions of the globe, namely South America and Australia where it is favored for meat production. Damaras have developed an innate ability to withstand seasonal weight loss [7] due to their evolution in a semi-arid environment that is prone to annual drought cycles. Previously we compared the muscle and liver proteomes between the Damara and Merino using proteomics and NMR metabolomics [8–10]. Results indicated that Damara sheep possess a unique fatty acid metabolism when compared to other breeds [6]. It involves oxidizing long-chain fatty acids into sterols that eventually are metabolized into glucose and ATP, a metabolic process that is present but limited in both humans and other breeds of sheep [8]. Furthermore, on our previous proteomics studies on this issue and involving the liver, we have found noteworthy differences between the two breeds with numerous biochemical pathways been affected in both liver and muscle [8,9]. Indeed, the majority of the identified pathways for their biological processes present in the comparisons that involve the Damara suggest that fat reserves are being converted to ATP via phosphorylation. This process is present in both breeds [8]. Results suggest however that this process is less down-regulated in Damaras allowing them to readily utilize fat reserves located within their tails, a feature that Merinos do not possess [8]. On the muscle side, we have studied SWL tolerance and proposed several proteins (S100-A10 Serpin A3-5-like and Catalase) that could be proposed for markers of SWL tolerance [9].

The liver is central to metabolism, particularly in aspects concerning energy sparing and spending mechanisms. Additionally, hepatic mitochondria are unique because they play a key pivotal role in carbohydrate, lipid and protein metabolism. In order to better understand the unique energy metabolism of Damara sheep, by comparison to Merino, Blue Native Electrophoresis (BNE) and two-dimensional electrophoresis (2DE) coupled to mass spectrometry (MS) were utilized to produce a 2DE and BNE mapping of the ovine hepatic mitochondria. This study provides a comparative analysis of the mitochondrial proteome between Damara and Merino sheep breeds with differing SWL tolerance and make sense of the mitochondria’s proteomic response in correlation to nutritional stress.

## Results and discussion

### Solubilization of the mitochondrial membrane protein complexes

**Fig 1:**
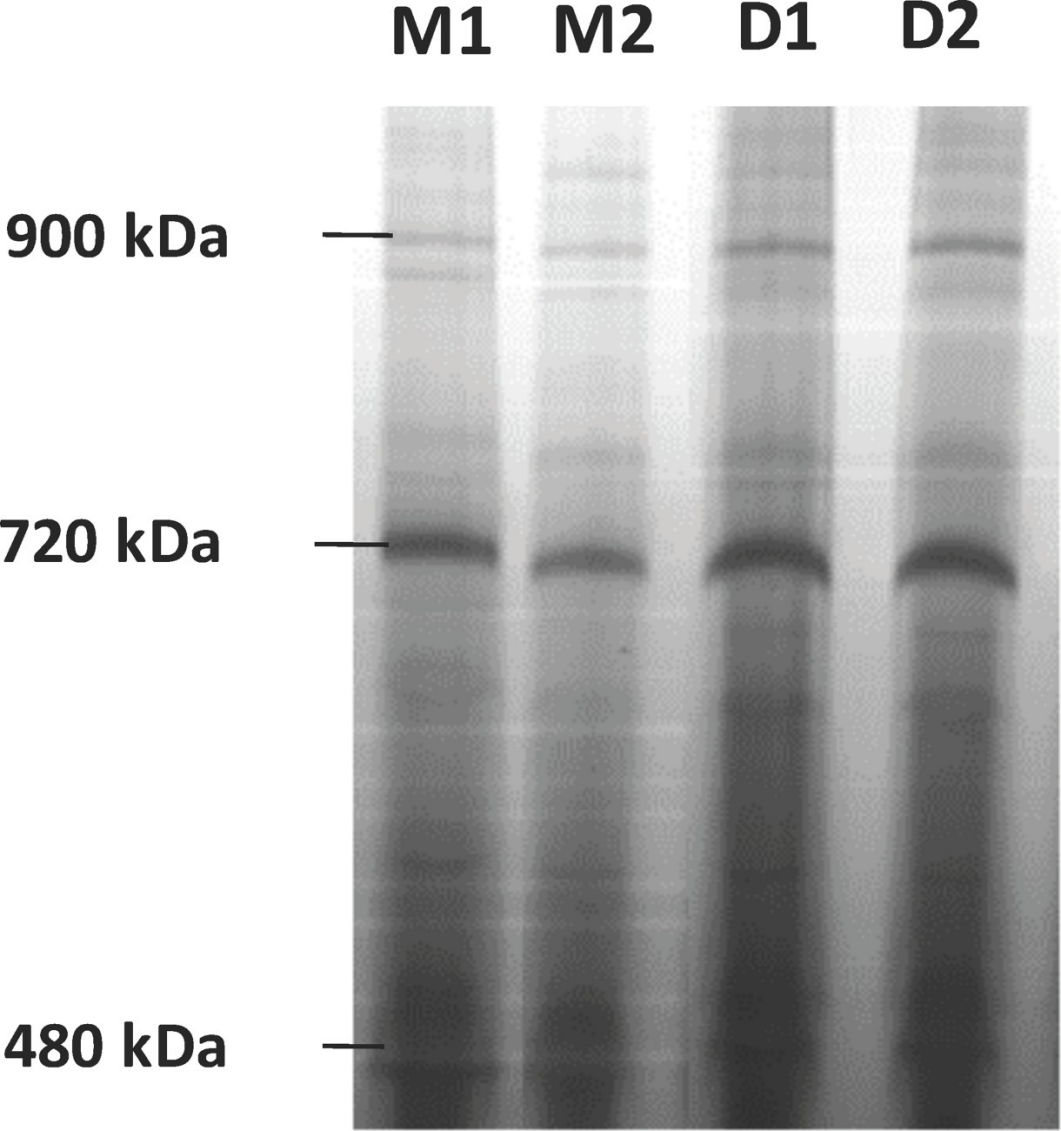
Separation of mitochondrial protein complexes by BN-PAGE by breed and diet (M = Merino, D = Damara, 1 = Restricted, 2 = Unrestricted). Proteins were solubilized with the detergent digitonin at 2% w/v. Gel stained with Coomassie Blue Colloidal. All gel bands at 480 kDa were excised and analyzed by nano-LC-MS/MS.

The efficiency of the solubilisation was assessed by protein profiling in BN-PAGE gels (number of bands and intensity). Upon electrophoresis, similar profiles were reported for the three protein samples tested with at least eight protein complexes being resolved among samples (Fig 1). The molecular mass of the protein complexes in the BN-PAGE gels varied between 200 kDA and 1,236 kDa. The results indicate that digitonin, within this concentration range has no major influence in the solubilisation of membrane proteins.

### BN/SDS PAGE of mitochondrial proteins

**Fig 2:**
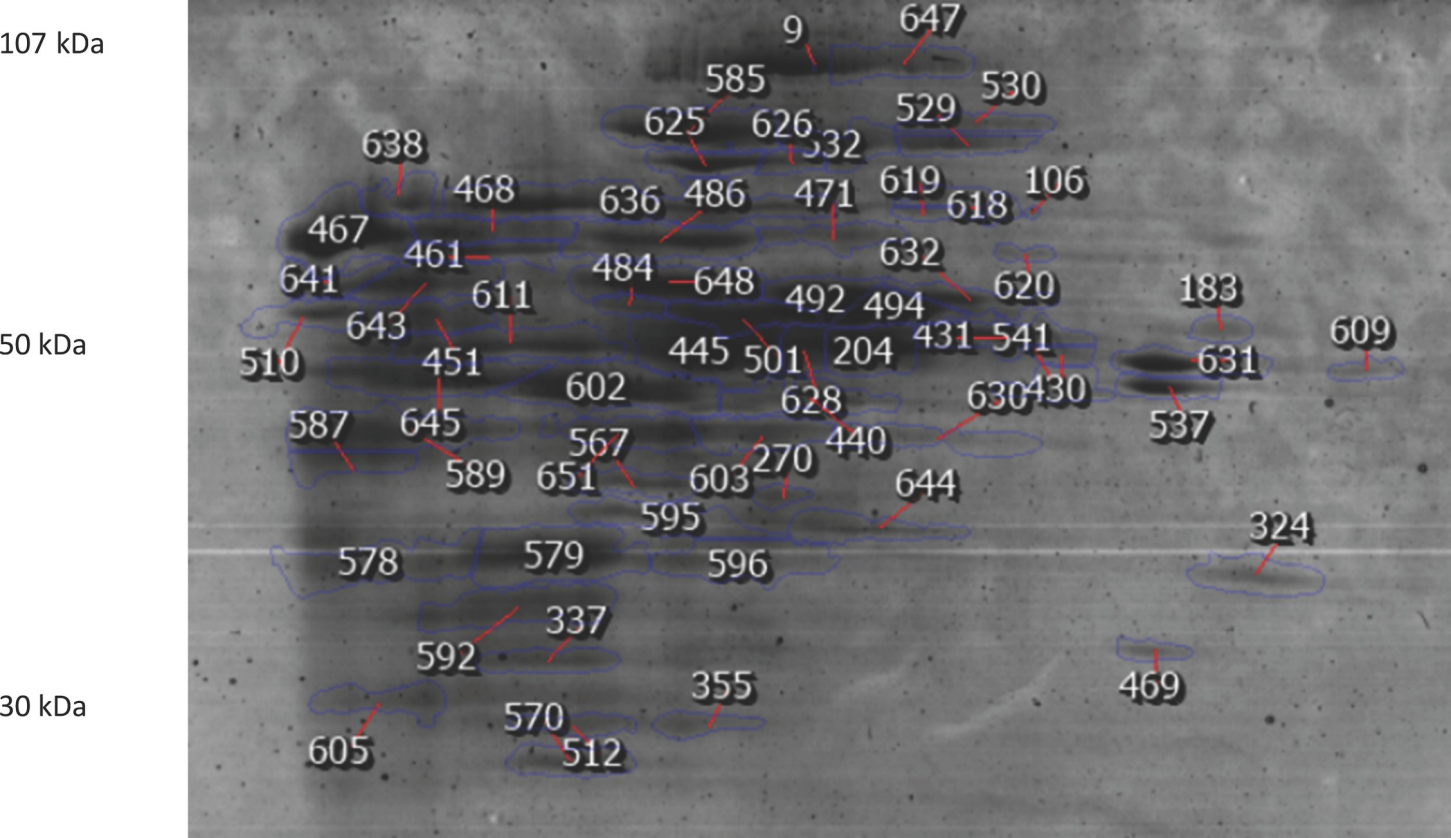
An example of one of the second dimension separations BN-PAGE / SDS-Page. Numbered protein spots were identified and detailed in Table 1. Proteins were stained with CCB and identified with MALDI-TOF/TOF.

2DE was employed for a complementary mapping of the mitochondrial proteome (Fig 2). In comparison to BN-PAGE on its own, the high resolution of 2DE allows for better description and thus easier characterization of protein isoforms [11]. This conventional proteomic analysis method enables the information here to be reproduced and utilized. Proteins with masses varying between 20 and 117 kDa were separated in large format gels along a pH interval of 3–10 (Fig 2). Kindly refer to S1 Report for further details.

### Proteomic analysis and gene ontology summary of identified proteins

Fifty proteins were identified total by LC-MS/MS with seven found to be differentially abundant between the two sheep breeds on two diets based on an ANOVA p-value. The majority of identified proteins were located in the mitochondrial membrane. Molecular functions for proteins include NADP+ activity, tRNA binding, angiostatin binding and calcium ion binding. Biological processes include toxin metabolism, epoxide metabolism, catalytic activity regulation, carbamoyl phosphate biosynthesis, protein lipidation and apoptosis.

### Differential proteomics

**Fig 3:**
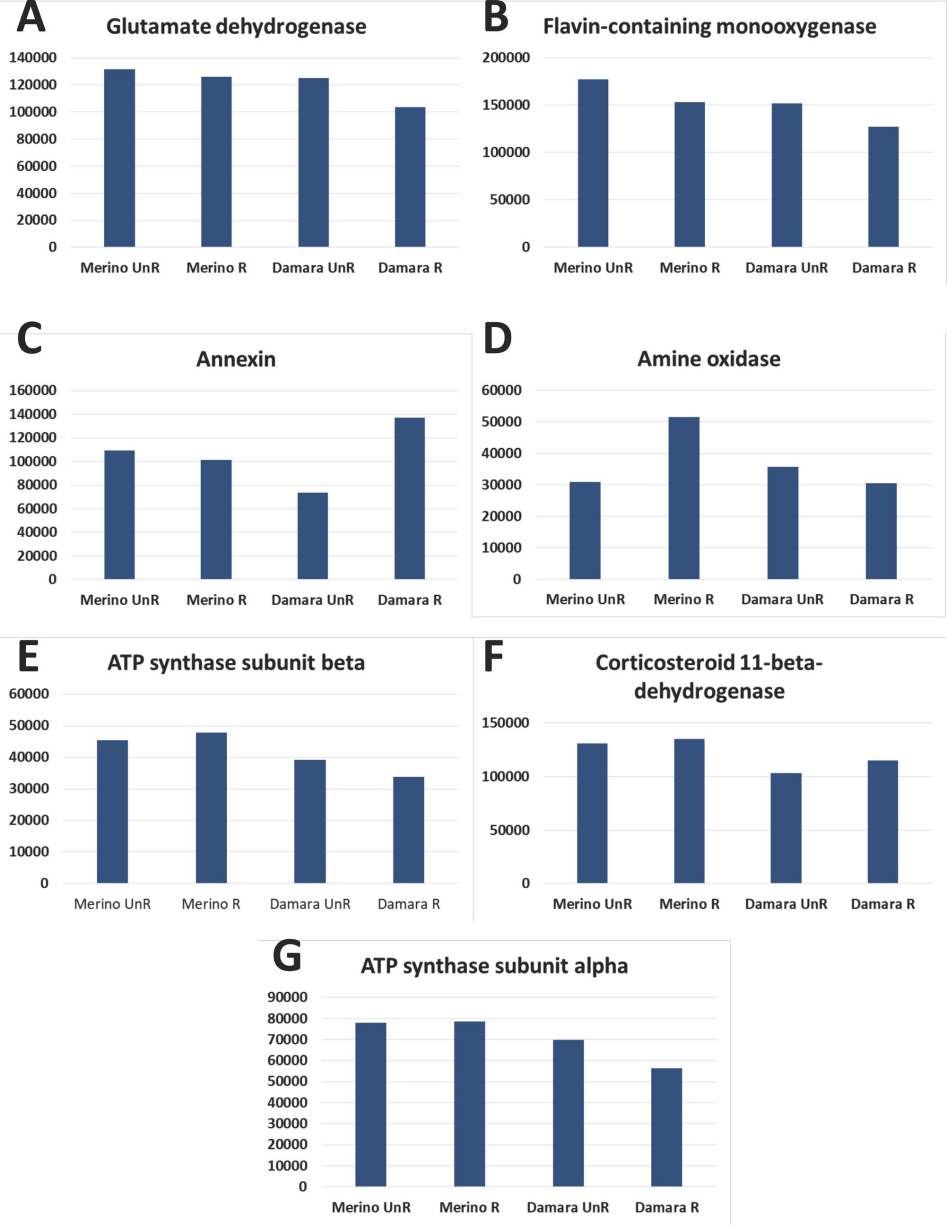
Expression profiles of the seven proteins showing differential abundance. Results shown as Log Normalized Volume. Please refer to S1 Report and S1 Table for further details and complete results. A—Glutamate dehydrogenase; B—Flavin-containing monooxygenase; C–Annexin; D–Amine Oxidase; E—ATP synthase subunit beta; F—Corticosteroid 11-beta-dehydrogenase and G—ATP synthase subunit alpha.

Quantification of all identified proteins via 2DE between the four experimental groups are presented in Table 1 with differentially abundant proteins highlighted. Protein changes in abundance between the varying breeds and treatments are detailed in Fig 3; these four comparisons are normalized quantifications between treatments expressed as a percent increase or decrease and so form the foundation for discussion of the proteins’ changes in abundance between treatments and breeds and their probable functions. A total of seven spots were quantified to have significant differential abundance based on an ANOVA test, allowing for quantification of seven differentially abundant protein spots from among the fifty identified: glutamate dehydrogenase, flavin-containing monooxygenase, annexin, amine oxidase, ATP synthase subunit alpha, ATP synthase subunit beta, and corticosteroid 11-beta-dehydrogenase isozyme 1. Their abundance patterns as they were correlated to dietary treatment and differing breed are discussed below.

**Table 1:** Hepatic mitochondrial proteome compiled from each of the 6 replicates by sheep breed and diet as determined via 2D-BN/SDS-PAGE coupled to LC-MS/MS.

Spot	Protein Name	Merino		Damara			
		Unrestricted	Restricted	Unrestricted	Restricted	Sum PEP Score	# of peptides
9	**Uncharacterized protein**	407.153	246.235	306.218	272.478	175.378	54
183	**Propionyl-CoA carboxylase beta subunit**	8645.38	9860.516	8433.862	8259.056	54.252	16
*204	**Glutamate dehydrogenase**	131800	126000	125200	103600	242.896	33
270	**Glyceraldehyde-3-phosphate dehydrogenase**	4185.334	5879.308	4787.491	4260.301	32.061	10
324	**Regucalcin**	25350	30640	30860	28120	67.538	14
337	**20beta-hydroxysteroid dehydrogenase type 2**	38880	32400	30060	26710	70.295	18
355	**Hydroxysteroid 17-beta dehydrogenase 10**	10620	10170	12080	10210	60.197	12
430	**Uncharacterized protein**	9794.302	8540.814	14210	8733.545	29.35	7
440	**Acetyl-Coenzyme A acyltransferase 2**	56010	47720	44940	48870	78.991	13
*445	**Flavin-containing monooxygenase**	177400	153500	151800	127300	51.285	15
451	**Cytochrome P450**	31350	40790	33290	29780	52.642	20
461	**Annexin**	24190	36870	28030	25080	144.732	35
*467	**Annexin**	109600	101400	73500	137100	112.135	39
*468	**Amine oxidase**	30890	51440	35720	30460	34.881	13
469	**Voltage-dependent anion channel 1**	10620	10290	8250.19	6433.11	22.115	8
471	**Solute carrier family 25 member 13**	25420	22900	20910	21210	106.714	26
486	**Succinate dehydrogenase**	71020	69210	69870	66100	64.535	16
492	**Carboxylic ester hydrolase**	127700	119900	103000	100700	185.961	28
494	**Uncharacterized protein**	85850	86180	86770	73830	146.606	29
512	**Enoyl-CoA hydratase**	13680	17180	12800	15670	42.737	10
529	**Aldehyde dehydrogenase 1**	25160	25750	33470	30420	54.38	23
530	**Nicotinamide nucleotide transhydrogenase**	15280	20130	20140	16830	109.468	25
532	**Glycogen phosphorylase**	36600	42620	40480	42990	85.811	37
*537	**ATP synthase subunit beta**	45370	47780	39250	33830	224.445	30
541	**Glutamate dehydrogenase**	17460	13830	20160	18190	60.499	19
570	**Hydroxysteroid 17-beta dehydrogenase 10**	23410	25820	21900	19420	74.725	13
578	**Uncharacterized protein**	85080	67340	61420	89110	42.328	13
*579	**Corticosteroid 11-beta-dehydrogenase**	131000	135400	103500	114800	77.613	18
585	**Methylenetetrahydrofolate dehydrogenase**	90060	96290	94220	87780	85.783	24
587	**Uncharacterized protein**	21700	24630	19120	25490	43.164	14
589	**Actin gamma 1**	96880	105400	73530	110600	131.039	18
592	**Hydroxysteroid 17-beta dehydrogenase**	69620	70650	56770	59720	60.356	13
595	**Fructose-1,6-bisphosphatase 1**	23590	31810	28370	27840	50.843	14
596	**Delta-aminolevulinic acid dehydratase**	40400	39340	46890	53730	22.538	10
602	**Epoxide hydrolase**	132700	115000	131900	120100	82.117	23
603	**Acetyl-Coenzyme A acyltransferase 2**	29570	39720	38390	39940	97.651	18
605	**Uncharacterized protein**	19020	20150	15660	19730	28.943	11
609	**ATP synthase subunit alpha**	7994.689	7170.036	7529.875	6504.324	75.815	19
611	**ATP synthase subunit alpha**	67770	77700	66360	60740	45.364	16
619	**Hydroxyacyl-coenzyme A dehydrogenase**	4342.836	3931.593	4474.777	4476.312	67.458	18
625	**Microsomal triglyceride transfer protein**	31420	32950	31830	33910	182.968	42
626	**Glycogen phosphorylase**	10090	9221.281	11600	11240	123.845	32
628	**Glutamate dehydrogenase**	31340	32810	33420	29950	141.519	29
*631	**ATP synthase subunit alpha**	77840	78500	69770	56300	117.791	22
636	**Heat shock protein family A (Hsp70) member 5**	151700	126900	154400	136300	146.587	26
638	**Protein disulfide-isomerase**	23120	21270	21260	21730	47.049	16
641	**Serum albumin**	27730	29150	18740	32280	31.068	15
643	**Protein disulfide-isomerase**	47680	48760	50130	43660	138.871	30
645	**Uncharacterized protein**	84060	103600	67740	81210	56.707	14
648	**Acyl-CoA dehydrogenase**	101200	104000	100500	93430	60.947	17

*Highlighted proteins are those that changed (increase of decrease) in abundance (P<0.05) differentially among the four experimental groups. Results of protein expression shown as Log Normalized Volume. Please refer to S1 Report and S1 Table for further details.

Glutamate dehydrogenase is an enzyme present in the mitochondria of most eukaryotes. In humans, it converts glutamate to α-ketoglutarate, one of two ketone derivatives of glutaric acid, and ammonia [12]. It is found at high levels in mammalian livers, kidneys, brains and pancreases. It is a glutamate inhibitor and helps offset the effects of nutritional stress in the pancreas. Glutamate dehydrogenase is also known to promote the metabolism of glutamate and glutamine for generating ATP [13]. In a comparison between Merinos on the unrestricted diet versus other Merinos on a restricted diet, Glutamate dehydrogenase decreases by 4.40% in the latter. In a comparison between Damaras on an unrestricted diet versus other Damaras on a restricted diet there was a 17.25% decrease in the latter. These abundance patterns suggest the ammonia metabolic cycle decreases its activity during times of nutritional stress in the Damara more so than in the Merino. In humans and other better described mammals, glutamate dehydrogenase is used as a clinical measure of liver function. Evidence demonstrates both breeds have an attenuation in liver enzymatic function but when comparing the Merino and the Damara both on the same restricted diet, a 17.87% decrease is observed in the Damara. This decrease was less severe between both breeds on the same unrestricted diet, however, as the Damara’s saw a comparatively smaller 5.01% decrease over the Merino. It is also possible that the Damara decrease its liver activity in general, given this protein is a measurement of liver function in other species.

Amine oxidase is a well-known catalyst of oxidative cleavage of alyklyamines into aldehydes and ammonia [14]. In a comparison between Merinos on a restricted diet versus other Merinos on an unrestricted diet, Amine oxidase saw a 66.53% increase in the latter. In a comparison between Damaras on an unrestricted diet versus other Damaras on a restricted diet there was a 17.25% decrease observed in the latter. Additionally, it decreased in the Damara when compared against the Merino on an unrestricted diet by 15.64%. When both breeds were compared on a restricted diet, the Damara saw a 40.79% decrease versus the Merino. Glutamate dehydrogenase, the previously mentioned protein, is also an ammonia metabolic protein like Amine oxidase. Amine oxidase, however, shows more variable behavior between breeds than Glutmate dehydrogenase. When both are placed in context together, it is clear that both breeds demonstrate changes in ammonia metabolic activity in response to nutritional stress. However, in the Damara, both proteins, Amine oxidase and Glutamate dehydrogenase are affected in the same negative direction, where the Merino displays abundances of these two enzymes very differently from each other.

Flavin-containing monooxygenases are proteins that are specialized in the oxidation of xenobiotic factors for extraction and elimination of non-nutritional and insoluble compounds [15]. Such enzymes can oxidize soft nucleophiles and sulfides. Flavin-containing mono-oxygenases decreases in a comparison of the Merino on an unrestricted diet versus other Merinos on a restricted diet by 13.47% in the latter. The Damara on an unrestricted diet versus other Damaras on the restricted diet saw a similar 16.14% decrease between dietary treatments. While abundance levels are similar in both breeds, the Damara showed a 14.43% decrease on the unrestricted diet versus Merinos on the unrestricted diet and a 17.07% decrease on the restricted diet versus the Merino on the restricted diet. In a previous study utilizing different tissue from the same animal carcasses, muscle proteins were associated with xenobiotic metabolic processes [8]. Furthermore, flavin-containing monooxygenases are frequently grouped with and misclassified as members of the cytochrome p450 group due to structural similarities between the two types of proteins; namely an NADPH binding domain, FAD binding domain and conserved arginine residue [16]. Significantly more Cytochrome p450 was previously found in the liver of the Damara compared to that of the Merino in the aforementioned study [8]. It is of note that such a closely related and often confused protein is lower in abundance in the liver mitochondria of the Damara on an unrestricted diet versus other Damaras on the restricted diet when its cytochrome p450 abundances are so high in the liver. Both proteins are implicated in multiple fatty acid, sterol and inflammation system biological processes, corroborating previous results pointing to the Damara having a unique fatty acid metabolism.

As described [17], Annexin is another fatty acid metabolic protein is predominantly found inside the cells of eukaryotes, being excreted into blood. Annexin suppresses phospholipase A2, an enzyme that ultimately results in eicosanoids production [17]. Eicosanoids are derived from fatty acids and include inflammation mediators, with the Annexin-1 gene playing a major role in this process. In our study, the Merino on an unrestricted diet versus other Merinos on a restricted diet demonstrates a 7.78% decrease in Annexin. In a comparison between the Damara on an unrestricted diet versus other Damaras on a restricted diet, there is an 86.53% increase in the latter. In a comparison of the Merino on the unrestricted diet versus Damaras on the unrestricted diet, a 32.49% decrease was observed in the latter. In a comparison of the Merino versus the Damara on the restricted diet, there is a 35.21% increase in the latter when both breeds’ proteome response are compared. The Damara’s unique fatty acid metabolic process could affect the production of corticosteroid-associated gene products and metabolites. Fatty acid metabolic proteins described in our previous study on the liver proteome using shotgun proteomics demonstrated important changes between breeds for instance for proteins such as fatty acid desaturase 2 or fatty acid synthase isoform X2 [8]. Lipid metabolism proteins have been linked to inflammation control in dairy cows, as seen in a recent review by Ceciliani and co-workers [18]. The Damara is known to be very efficient in mobilizing the adipose tissue located in its tail when under nutritional stress. It can therefore be suggested that Annexin is a protein that is likely linked with both mobilizing fat reserves more readily during times of nutritional stress in conjugation with the previously described immune response [19], therefore playing a major role in regulating the inflammation system, allowing these animals to cope better with SWL-induced stress. This provides evidence to suggest that Damara sheep have a unique inflammation response. Furthermore, fatty acid metabolites, sterol metabolites, corticosteroid metabolites share metabolic precursors with fatty acids, suggesting there are likely additional concurrent increases and decreases in inflammation function happening related to the Damara’s unique fatty acid metabolism. Finally, Annexin’s abundance patterns between breeds along gives it a promising start as a potential future biomarker of tolerance to SWL at the liver level that could be further investigated.

Corticosteroid 11-beta-dehydrogenase isozyme 1 is an enzyme that catalyzes conversion of cortisol to cortisone. Levels were relatively unchanged in a comparison of the Merino on an unrestricted diet versus other Merinos on a restricted diet with 3.36% increase in the latter. In a comparison of the Damara on an unrestricted diet versus other Damaras on a restricted diet, there was a 10.92% decreases in the latter. The Damara has consistently lower levels regardless of dietary treatment (a 20.10% decrease in the Damara on an unrestricted diet versus the Merino and a 15.21% decrease in the Damara on a restricted diet versus the Merino). Cortisol, of which corticosteroid 11-beta hydrogenase isozyme 1 is a catabolizer, is well documented to spike under conditions of nutritional stress, leading to both acute and chronic inflammation. Corticosteroid 11’s behavior here suggests that the Damara reduces its cortisol response to nutritional stress by producing less corticosteroid and thus produces less cortisol overall. This would leave the Damara in a state of lower inflammation than the Merino, allowing it to remain more productive on a restricted diet by not diverting so many resources to managing chronic inflammation during dry-seasons. This protein’s behavior suggests similar inflammation / fatty acid metabolic overlap in the Damara as Annexin does.

ATP synthase subunit alpha and ATP synthase subunit beta are two related proteins found to be differentially abundant in our study that together form a membrane bound enzyme complex implicated in ATP synthesis using oxidative phosphorylation. It is typically performed across the inner mitochondrial membrane; deletion of the gene associated with the protein in mice results in decreased ATP levels, particularly following nutritional stress [20,21]. In a comparison of Merinos on the unrestricted diet versus other Merinos on a restricted diet, a slight increase was found in the latter (0.85% and 5.31% for alpha and beta respectively). In a comparison of the Damara on the unrestricted diet versus other Damaras on the restricted diet, both ATP synthase sub-units alpha AND beta demonstrates a significant drop in abundance in the latter (19.31% and 13.81% respectively). The Damara also shows decreases for both alpha and beta versus the Merino on both diets (10.37% and 13.49% respectively for the unrestricted comparison and 28.28% and 29.20% for the restricted comparison respectively) Lower levels of ATP synthase subunits may indicate the Damara utilizes additional pathways of energy production than the Merino, perhaps through greater metabolism of adipose tissue to utilize fatty acids during times of nutritional stress as opposed to carbohydrates [6].

### Future considerations for each differentially abundant protein

Glutamate dehydrogenase to liver liver function in mammals [22]. More “traditional” less cost-prohibitive immuno-assays could be used if a suitable anti-body for amine oxidase can be found for sheep. Measuring liver function of both breeds directly in much larger numbers would shed considerable light on this phenomenon.

Amine oxidase (along with glutamate dehydrogenase) both suggest significant differences in activity in the urea cycle between both breeds as well. A proteomic approach (or similar high throughput technique for assessing whole systems responses) exploring the Damara’s kidney function versus a breed like the Merino would be a good starting place for further exploration of adaptations to SWL and likely provide insight into its hydration and fluid intake management. Given the evolutionary demands of arid climates, it is likely the Damara displays unique function, particularly where management of waste like ammonia is concerned.

Flavin containing mono-oxygenase is closely associated with cytochrome p450, a protein that was described in a previous study to behave in abundance patterns that are inverse to flavin containing mono-oxygenase. This warrants future study and suggests further validation.

While many fatty acid proteins often possesses significant inflammation system function as well under its list of gene ontology terms, Annexin in particular speaks to a fatty acid metabolic and immune system overlap of protein function and has abundance patterns that make it the strongest candidate as a potential biomarker to come out of this particular study.

Both ATP synthsase subunits alpha and beta are lower in the Damara than the Merino and further reinforce that the Damara’s nutritional requirements are just not standard to other more typical breeds of sheep. Future studies looking specifically into ATP metabolism as a whole, with metabolite and clinical biochemistry employed to generally try and assess what differentiates the Damara from the Merino in energy metabolism should be considered.

### Final conclusions

As stated previously, we suggest a link between the inflammation system and various fatty acid derived sterol and lipid metabolites. These results corroborate that, compared to the Merino, Damara sheep have not only a distinct fatty acid metabolism that allows efficient mobilization of adipose tissue to combat nutritional stress but also a unique inflammation and stress-pathway response. This likely helps prioritize the mobilization of fatty acid reserves and keep homeostasis during the dry season. In light of both the fatty acid and inflammation-system response results, future considerations should be given to the Damara’s inflammatory response when compared to other breeds. The connection between digesting fatty acid metabolites and the inflammation system could also be considered.

This study marks the first description of the proteome of *Ovine* hepatic mitochondria. Proteins were first mapped utilizing BN-PAGE followed by 2DE as descriptive tools. The proteomic profiles that resulted demonstrates continued evidence that the Damara is uniquely suited to withstand seasonal weight loss compared to the Merino.

Finally, the ontology of differentially abundant proteins points to unique inflammation physiology in the Damara with concurrent functions in sterol and lipid based metabolisms. While all seven differentially abundant proteins are candidates for subsequent validation, this study as well as similar studies form the basis for selection indices and all could putatively be quantified in different breeds with that role in mind. Following further validation, these protein indices could eventually be refined into single, easily identifiable biomarkers that can be utilized for selective breeding to produce an animal that is both more productive and tolerant to SWL, therefore contributing to solve this important issue in the context of ruminant production.

## Materials and methods

### Liver tissues and experimental design

Archived liver samples from a previous study were utilized [9, 23,24]. Briefly, Damara and Merino sheep were fed unrestricted and restricted diets for 42 days. Samples from a total of 24 sheep (12 Damara and 12 Merino) that were randomly allocated to either an unrestricted diet (simulated feed-rich pasture), or a restricted diet (simulated feed-poor pasture), were assessed. At the end of the 42 day period, animals were slaughtered in a commercial slaughter house and liver samples were taken and stored in -80°C until further analysis.

### Animal welfare disclaimer

All work involving animals was conducted according to relevant international guidelines that regulate the use of production animals in animal experimentation in the European Union (European Union procedures on animal experimentation–Directive 2010/63/EU) and Australia (Commonwealth and Western Australia state laws). This experiment was conducted with the approval of the Ethics Committee of the Department of Agriculture and Food Western Australia (DAFWA, Perth, WA, Australia) registered as process 07ME06. The entire trial was conducted under the supervision of the veterinary authority in the State of Western Australia. Author AM Almeida holds a FELASA (Federation of European Laboratory Animal Society Associations) grade C certificate that enables designing and carrying out animal experimentation under European Union regulations. Animal management, handling, transport and slaughter were all conducted replicating approved standard commercial practices in the Commonwealth of Australia and in the State of Western Australia. Animals included in this experiment were therefore subjected to the same welfare conditions as production animals.

### Mitochondrial extraction

Methodology for hepatic mitochondrial extraction has been previously described [25]. Briefly, liver samples were sectioned into small pieces and mixed with sucrose (0.25 M), EDTA (1.0 M), Hepes (10 mM of 2-[4-(2-hydroxyethyl)piperazin-1-yl]ethanesulfonic acid), pH 7.4 at 0.1g fresh weight tissue / 0.25ml buffer. The tissue mixtures were homogenized with mortar and pestle for 3 min, and then sonicated for 3 cycles at 60 Hz during 5s (VibraCell 50-sonics & Material Inc. Danbury, CT, USA). Mitochondrial fractions were obtained by differential centrifugation. The homogenate was first centrifuged for 10 min, at 1000x*g* at 4°C to discard cell debris. A second centrifugation for 25 min at 20000x*g* at 4°C was performed to obtain a mitochondrial pellet. The pellet was re-suspended and subjected to a second centrifugation at 20000x*g* to wash away cytosolic protein contaminants. Mitochondria pellet was re-suspended in commercial BN-PAGE sample buffer (Thermofisher, BN2003) at 0.1g fresh weight sample tissue per 0.25ml buffer. Protein concentrations within samples to be used on Blue Native PAGE were determined using the Bradford Method [26]. Extracts were stored at -80°C until further analysis.

### Sample preparation and BN-PAGE/SDS-PAGE

Mitochondrial extract samples were diluted to 4 mg protein/ml in BN-PAGE sample buffer. Digitonin detergent was added to the samples to a final concentration of 2% (w/v) to enable solubilization of mitochondrial protein complexes. The samples were incubated with digitonin for 30 min in ice, and then centrifuged for 20 min at 20000 g and 4°C. Coomassie G-250 was added to the sample at 1/4^th^ of the detergent concentration from a commercial G-250 stock solution (Thermofisher, BN2003) before loading the samples in the BN-PAGE gel. Protein complexes were separated by BN-PAGE employing the commercial NativePAGE Novex Bis-Tris gel system. A total of 800 μg protein was loaded in each well, and proteins were next separated in gradient BN-PAGE gels (3 to 12%, w/v, acrylamide) (NativePage gels, Themofisher, BN1003BOX) at 150V. 2DE was employed for a complementary mapping of the mitochondrial proteome (Fig 2). In comparison to BN-PAGE on its own, the high resolution of 2DE allows for better description and thus easier characterization of protein isoforms [11]. This conventional proteomic analysis method enables the information here to be reproduced and utilized. Proteins with masses varying between 20 and 117 kDa were separated in large format gels along a pH interval of 3–10 (Fig 2).

To carry out the second dimension electrophoresis (SDS-PAGE), BN-PAGE lanes were isolated, incubated in 2% SDS (w /v), 66 mm Na_2_CO_3_, 0.67% β-mercaptoethanol (β-ME, v/v) for 20 min to reduce proteins, and immediately assembled in the top of a SDS-PAGE gel (12% acrylamide). Proteins were separated at 150 V. Protein bands (BN-PAGE) and spots (BN/SDS-PAGE) were visualized with colloidal Coomassie blue (CCB). Gel images were acquired with a GS-800 calibrated scanner (Bio-Rad, Hercules, CA, USA) and protein patterns analyzed and quantified prior to excision via spot intensity with the Samespots software (GE Healthcare, Newcastle, UK). An ANOVA p-test was performed to check each spot for significant variance in abundance between the four treatments. Differences in abundance between groups were then normalized to percent difference instead of spot color intensity.

### Protein identification using LC-MS/MS

Peptide samples were next analyzed by nanoLC-MS/MS using an Ultimate 3000 liquid chromatography system coupled to a Q-Exactive Hybrid Quadrupole-Orbitrap mass spectrometer (Thermo Scientific, Bremen, Germany) as described [27]. Samples were loaded onto a trapping cartridge (Acclaim PepMap C18 100A°, 5 mm x 300 μm i.d., 160454, Thermo Scientific) in a mobile phase of 2% ACN, 0.1% FA at 10 μL/min. After 3 min loading, the trap column was switched in-line to a 15 cm by 75μm inner diameter EASY-Spray column (ES800, PepMap RSLC, C18, 3 μm, Thermo Scientific, Bremen, Germany) at 300 nL/min. Separation was generated by mixing A: 0.1% FA, and B: 80% ACN, with the following gradient: 22 min (2.5% B to 50% B), 5 min (50% B to 95% B), 10 min (hold 95% B). Data acquisition was controlled by Xcalibur 4.0 and Tune 2.8 software (Thermo Scientific, Bremen, Germany).

The mass spectrometer was operated in data-dependent (dd) positive acquisition mode alternating between a full scan (*m/z* 300–2000) and subsequent HCD MS/MS of the 10 most intense peaks from full scan (normalized collision energy of 27%). ESI spray voltage was 1.9 kV. Global settings: use lock masses best (*m/z* 445.12003), lock mass injection Full MS, chrom. peak width (FWHM) 15s. Full scan settings: 70k resolution (*m/z* 200), AGC target 3e6, maximum injection time 100 ms. dd settings: minimum AGC target 1e3, intensity threshold 1e4, charge exclusion: unassigned, 1, 5–8, >8, peptide match preferred, exclude isotopes on, dynamic exclusion 20s. MS2 settings: microscans 1, resolution 17.5k (*m/z* 200), AGC target 1e5, maximum injection time 100 ms, isolation window 2.0 *m/z*, isolation offset 0.0 m/z, spectrum data type profile.

The raw data was processed using Proteome Discoverer 2.2.0.388 software (Thermo Scientific) and searched against the UniProt database for the taxonomic selection *Ovies aries* (November 2017 release). The Sequest HT search engine was used to identify tryptic peptides. The ion mass tolerance was 10 ppm for precursor ions and 0.02 Da for fragment ions. Maximum allowed missing cleavage sites was set to 2. Cysteine carbamidomethylation was defined as constant modification. Methionine oxidation and protein N-terminus acetylation were defined as variable modifications. Peptide confidence was set to high. The processing node Percolator was enabled with the following settings: maximum delta Cn 0.05; decoy database search target FDR 1%, validation of based on q-value. Multiple proteins were identified per spot (again, see Fig 2 for the 2DE spot map). The most abundant protein of these ID’d proteins were determined by selecting the protein from the list with the highest sum-pep score as well as the number of peptides (minimum of 2) present; for a protein identification to be accepted it had to be successfully identified based on these criteria in all samples. These other identified proteins are detailed in S1 Table.

### Gene ontology summation of quantified proteins

Attempts were made to match quantified ovine proteins were matched to orthologues in humans, rats and an all mammal database. The majority of mitochondrial proteins from this experiment did not map with high confidence (e-value cutoff at < 1E-6). Because of this, whole ontologies for differentially abundant quantified proteins were retrieved from the database QuickGO.The ontologies retrieved from QuickGO were then entereted into the summary ReviGO were used to produce non-hierarchical summaries [28,29].

## Supporting information

S1 Report Gel software analysis report. (PDF)

S1 Table Protein expression and identification data. (XLSX)
