# Supplementary material for: The ovine hepatic mitochondrial proteome: Understanding seasonal weight loss tolerance in two distinct breeds
Source: PLoS One. 2019 Feb 20;14(2):e0212580. doi: 10.1371/journal.pone.0212580 (PMC6382166; doi:10.1371/journal.pone.0212580)

Merino v Damara

Experiment: Merino v Damara

Report created: 7/25/2017 4:35:54 PM

Reference image

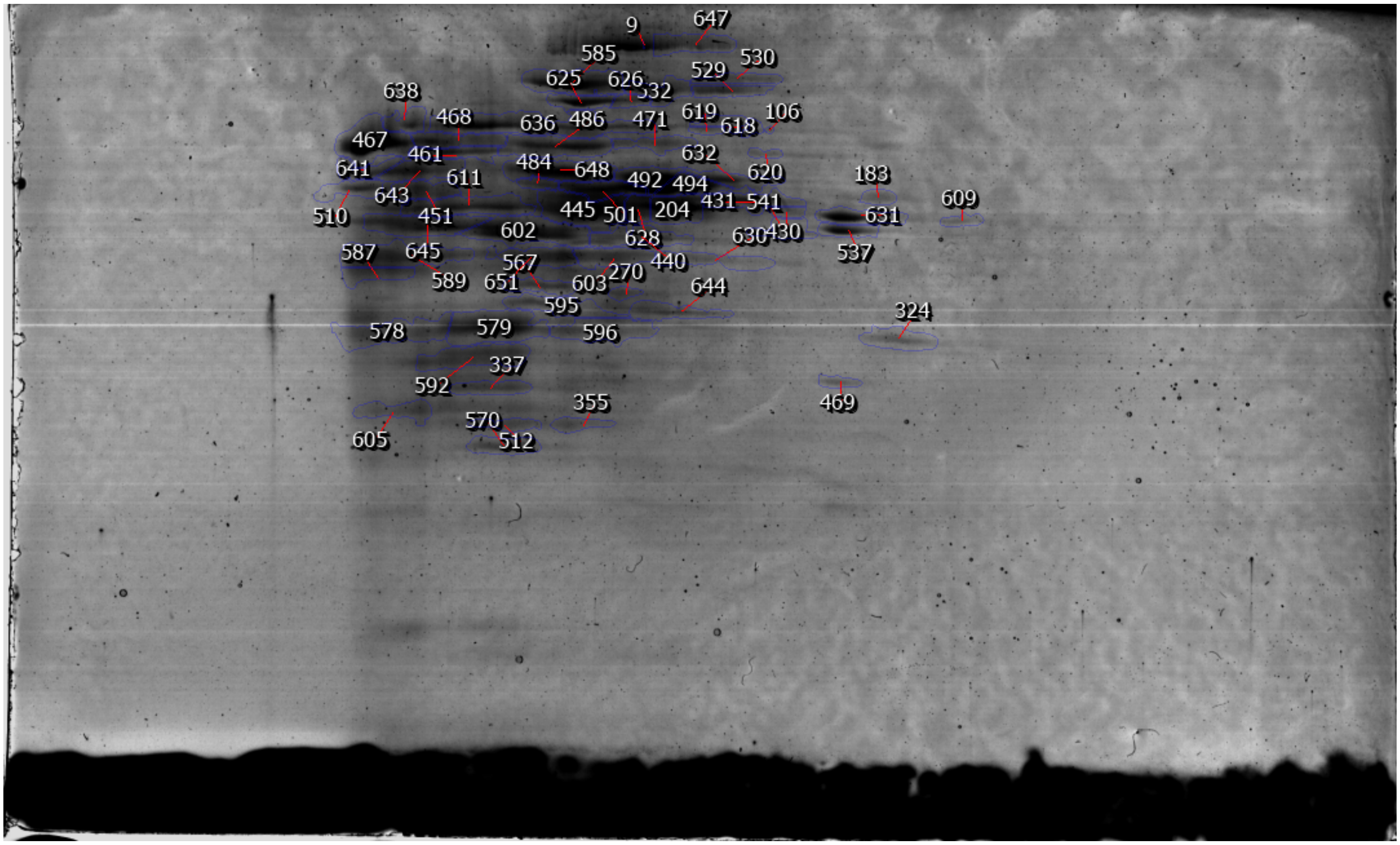

Experiment Design

Condition	D1	D2	M2	M1
Replicates	6	6	6	6

Spots

#	Anova (p)	Fold	Tags	Notes	pI	MW	Protein Accession	Protein Description	Protein pI	Protein MW	Protein URL	Average Normalised Volumes			
												D1	D2	M2	M1
204	0.004	1.3										1.036e+005	1.252e+005	1.318e+005	1.260e+005
537	0.006	1.4										3.383e+004	3.925e+004	4.537e+004	4.778e+004
631	0.007	1.4										5.630e+004	6.977e+004	7.784e+004	7.850e+004
579	0.007	1.3										1.148e+005	1.035e+005	1.310e+005	1.354e+005
445	0.016	1.4										1.273e+005	1.518e+005	1.774e+005	1.535e+005
461	0.025	1.5										2.508e+004	2.803e+004	2.419e+004	3.687e+004
430	0.025	1.7										8733.545	1.421e+004	9794.302	8540.814
468	0.028	1.7										3.046e+004	3.572e+004	3.089e+004	5.144e+004
467	0.031	1.9										1.371e+005	7.350e+004	1.096e+005	1.014e+005
510	0.047	1.9										5.501e+004	3.476e+004	6.503e+004	5.747e+004
641	0.051	1.7										3.228e+004	1.874e+004	2.773e+004	2.915e+004
469	0.063	1.7										6433.110	8250.190	1.062e+004	1.029e+004
595	0.065	1.3										2.784e+004	2.837e+004	2.359e+004	3.181e+004
431	0.083	1.3										5.322e+004	6.826e+004	6.373e+004	6.687e+004
596	0.089	1.4										5.373e+004	4.689e+004	4.040e+004	3.934e+004

#	Anova (p)	Fold	Tags	Notes	pI	MW	Protein Accession	Protein Description	Protein pI	Protein MW	Protein URL	Average Normalised Volumes			
												D1	D2	M2	M1
337	0.094	1.5										2.671e+004	3.006e+004	3.888e+004	3.240e+004
611	0.104	1.3										6.074e+004	6.636e+004	6.777e+004	7.770e+004
529	0.113	1.3										3.042e+004	3.347e+004	2.516e+004	2.575e+004
541	0.118	1.5										1.819e+004	2.016e+004	1.746e+004	1.383e+004
645	0.136	1.5										8.121e+004	6.774e+004	8.406e+004	1.036e+005
494	0.138	1.2										7.383e+004	8.677e+004	8.585e+004	8.618e+004
589	0.161	1.5										1.106e+005	7.353e+004	9.688e+004	1.054e+005
492	0.169	1.3										1.007e+005	1.030e+005	1.277e+005	1.199e+005
501	0.179	1.3										5.505e+004	5.114e+004	5.615e+004	6.604e+004
605	0.206	1.3										1.973e+004	1.566e+004	1.902e+004	2.015e+004
270	0.209	1.4										4260.301	4787.491	4185.334	5879.308
603	0.227	1.4										3.994e+004	3.839e+004	2.957e+004	3.972e+004
578	0.228	1.5										8.911e+004	6.142e+004	8.508e+004	6.734e+004
647	0.249	1.6										3.814e+004	4.213e+004	2.760e+004	4.523e+004
471	0.286	1.2										2.121e+004	2.091e+004	2.542e+004	2.290e+004
484	0.290	1.2										9559.317	8529.088	1.050e+004	1.046e+004
9	0.297	1.7										272.478	306.218	407.153	246.235
440	0.307	1.2										4.887e+004	4.494e+004	5.601e+004	4.772e+004
636	0.309	1.2										1.363e+005	1.544e+005	1.517e+005	1.269e+005
587	0.325	1.3										2.549e+004	1.912e+004	2.170e+004	2.463e+004
451	0.330	1.4										2.978e+004	3.329e+004	3.135e+004	4.079e+004
512	0.340	1.3										1.567e+004	1.280e+004	1.368e+004	1.718e+004
618	0.340	1.4										1.872e+004	1.727e+004	1.406e+004	2.032e+004
626	0.362	1.3										1.124e+004	1.160e+004	1.009e+004	9221.281
530	0.386	1.3										1.683e+004	2.014e+004	1.528e+004	2.013e+004
592	0.417	1.2										5.972e+004	5.677e+004	6.962e+004	7.065e+004
570	0.419	1.3										1.942e+004	2.190e+004	2.341e+004	2.582e+004
324	0.459	1.2										2.812e+004	3.086e+004	2.535e+004	3.064e+004
585	0.529	1.1										8.778e+004	9.422e+004	9.006e+004	9.629e+004
651	0.541	1.2										3.331e+004	3.777e+004	3.677e+004	4.056e+004
106	0.554	1.2										640.565	533.714	617.316	518.427
532	0.567	1.2										4.299e+004	4.048e+004	3.660e+004	4.262e+004
644	0.573	1.2										3.630e+004	3.270e+004	3.091e+004	3.671e+004
648	0.589	1.1										9.343e+004	1.005e+005	1.012e+005	1.040e+005
602	0.622	1.2										1.201e+005	1.319e+005	1.327e+005	1.150e+005
620	0.654	1.2										5172.250	4767.880	5207.887	4407.698
628	0.660	1.1										2.995e+004	3.342e+004	3.134e+004	3.281e+004
625	0.714	1.1										3.391e+004	3.183e+004	3.142e+004	3.295e+004
183	0.774	1.2										8259.056	8433.862	8645.380	9860.516
619	0.778	1.1										4476.312	4474.777	4342.836	3931.593
355	0.815	1.2										1.021e+004	1.208e+004	1.062e+004	1.017e+004
638	0.815	1.1										2.173e+004	2.126e+004	2.312e+004	2.127e+004
630	0.834	1.2										3.067e+004	3.275e+004	2.791e+004	3.081e+004
567	0.856	1.1										2.264e+004	2.311e+004	2.020e+004	2.144e+004
486	0.876	1.1										6.610e+004	6.987e+004	7.102e+004	6.921e+004
632	0.876	1.1										3.449e+004	3.501e+004	3.713e+004	3.443e+004
609	0.905	1.2										6504.324	7529.875	7994.689	7170.036
643	0.951	1.1										4.366e+004	5.013e+004	4.768e+004	4.876e+004

Tags	
	Edited
	Anova p-value ≤ 0.05

Identifier 204

Position (1026, 309)

Notes

Anova p-value ≤ 0.05

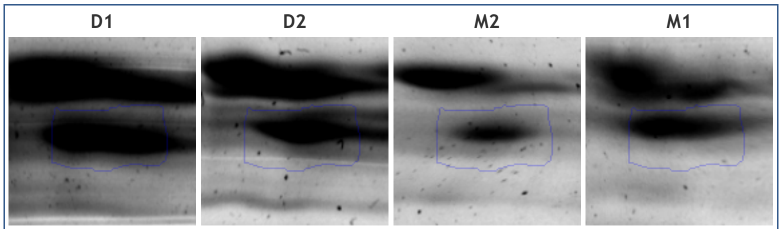

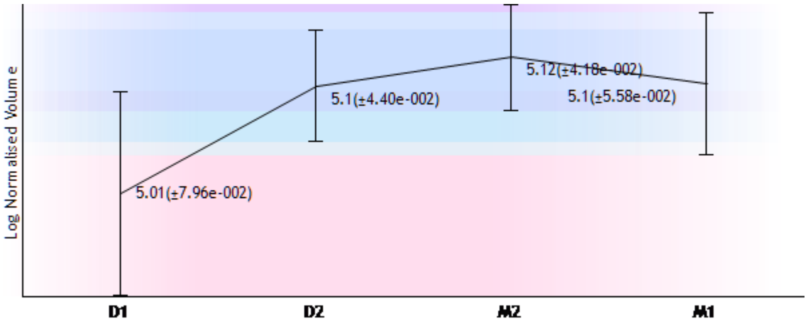

Identifier 537

Position (1284, 352)

Notes

Anova p-value ≤ 0.05

Edited

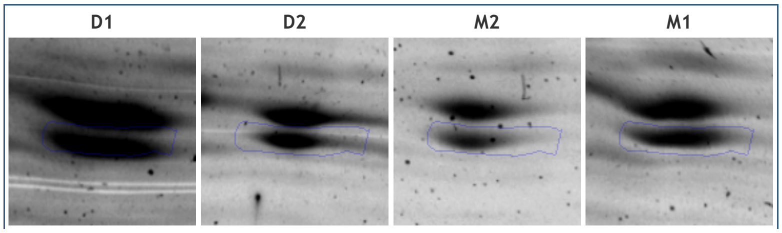

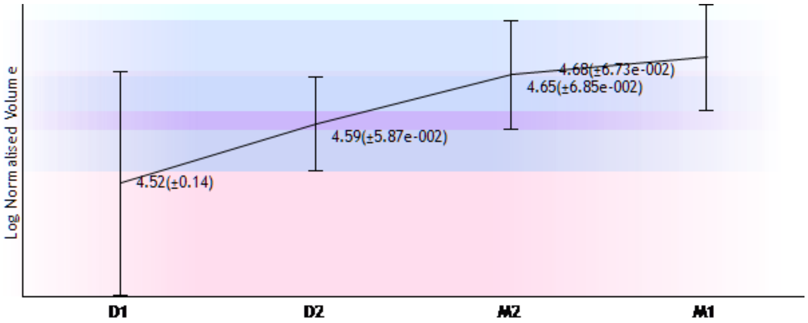

Identifier 631

Position (1280, 329)

Notes

Anova p-value ≤ 0.05

Edited

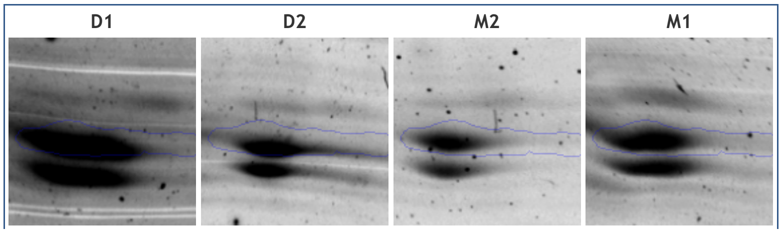

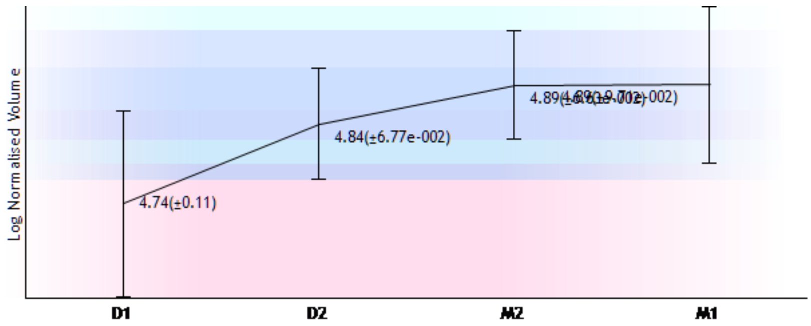

Identifier 579

Position (741, 495)

Notes

- Anova p-value ≤ 0.05
- Edited

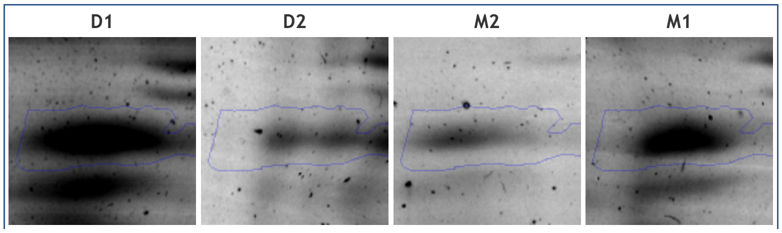

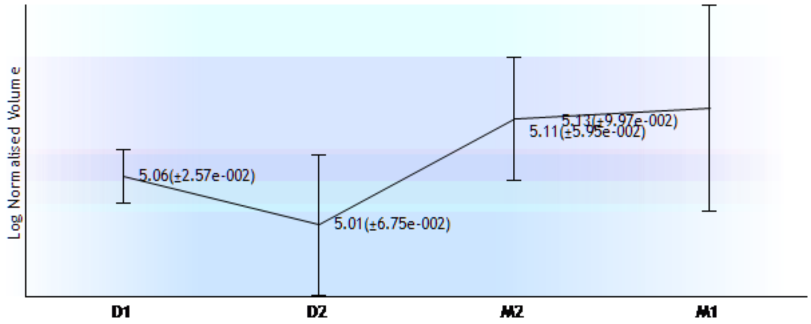

Identifier 445

Position (874, 327)

Notes

- Anova p-value ≤ 0.05
- Edited

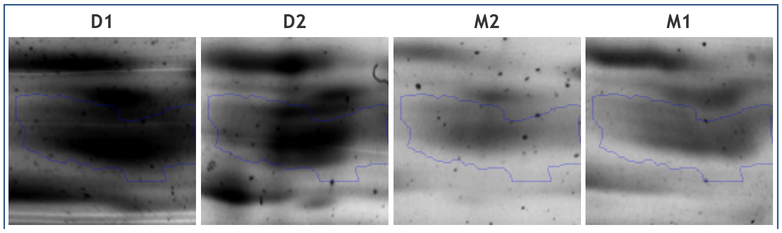

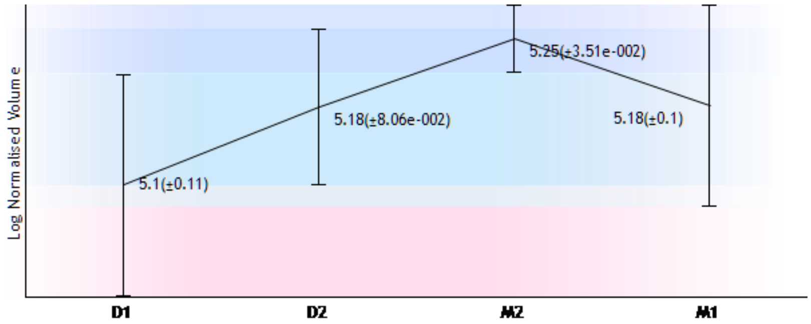

Identifier 461

Position (709, 228)

Notes

- Anova p-value ≤ 0.05
- Edited

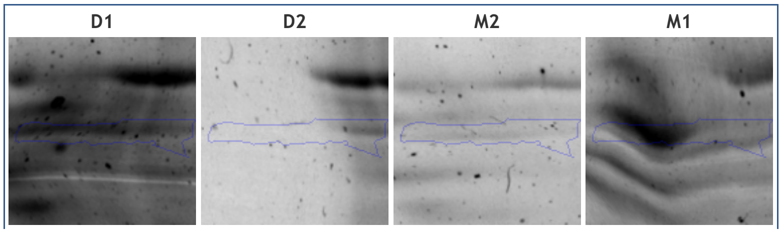

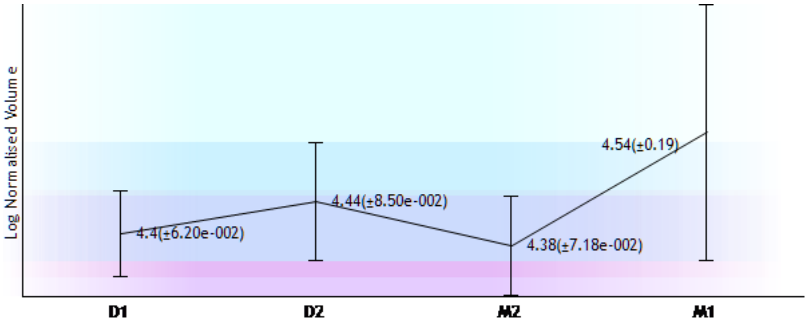

Identifier 430

Position (1184, 309)

Notes

- Anova p-value ≤ 0.05
- Edited

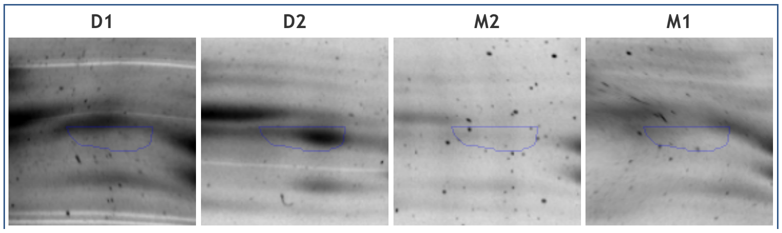

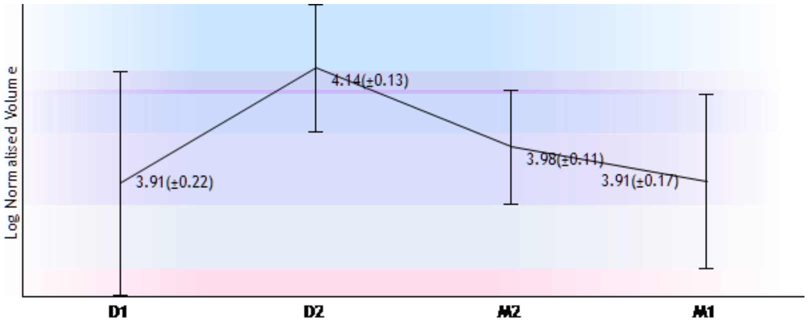

Identifier 468

Position (648, 201)

Notes

- Anova p-value ≤ 0.05
- Edited

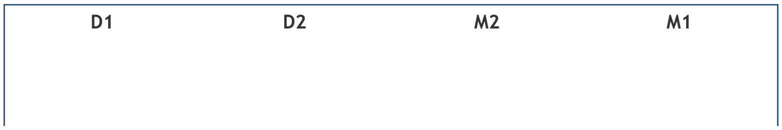

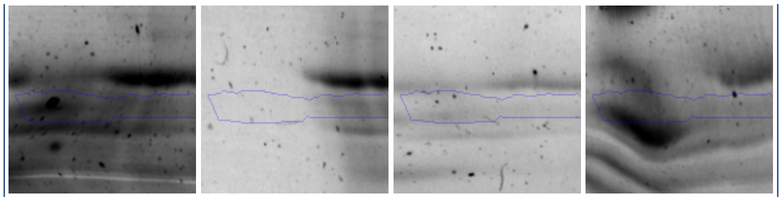

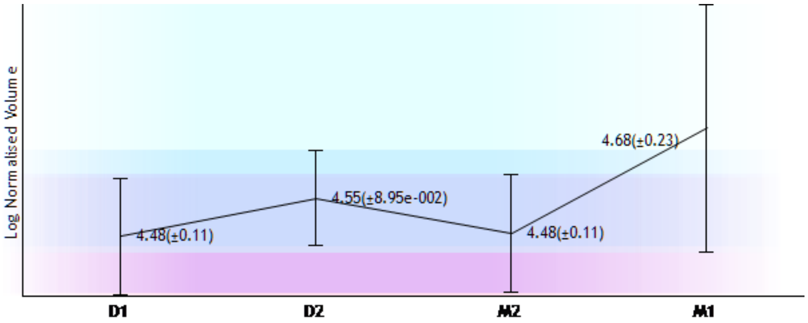

Identifier 467

Position (554, 224)

Notes

- Anova p-value ≤ 0.05
- Edited

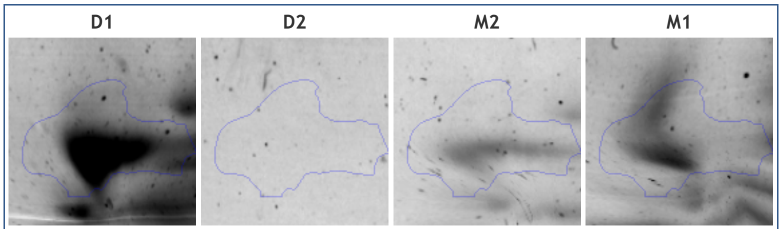

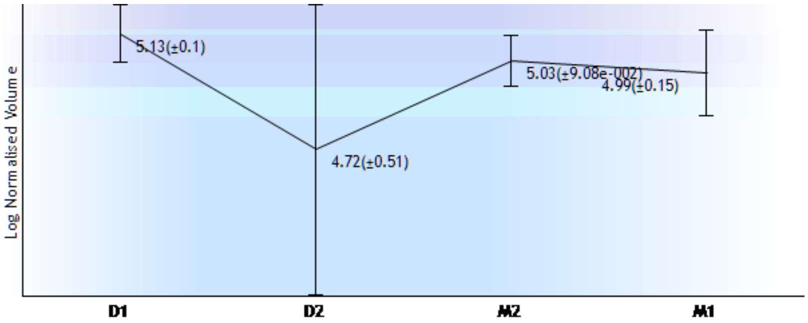

Identifier 510

Position (540, 279)

Notes

- Anova p-value ≤ 0.05
- Edited

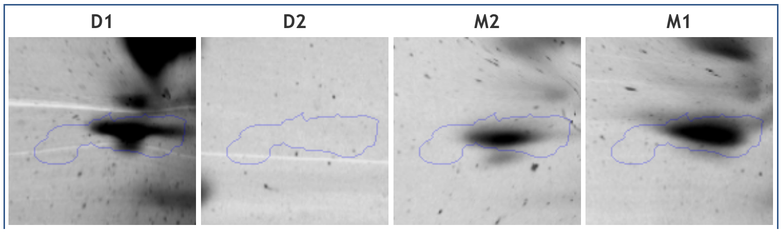

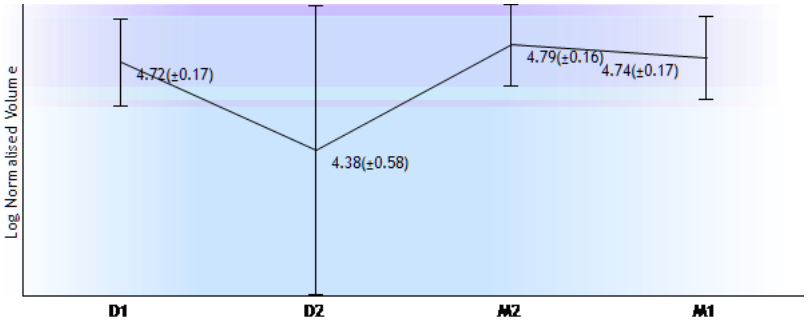

Identifier 641

Position (540, 256)

Notes

Edited

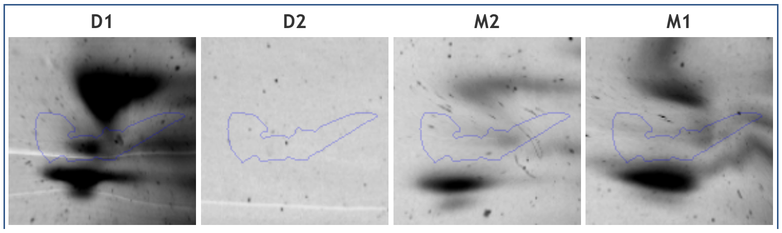

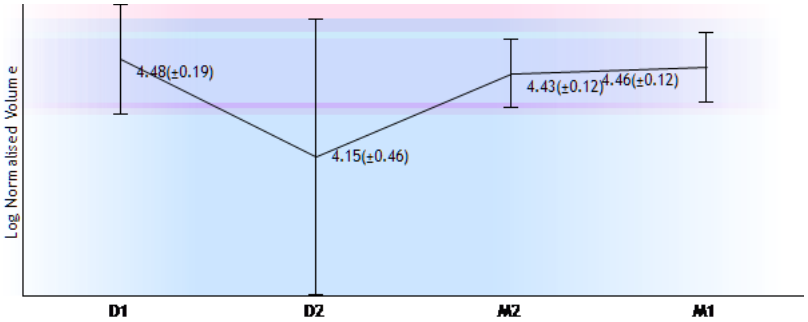

Identifier 469

Position (1248, 573)

Notes

Edited

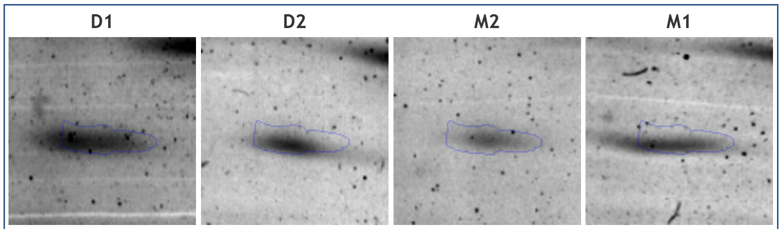

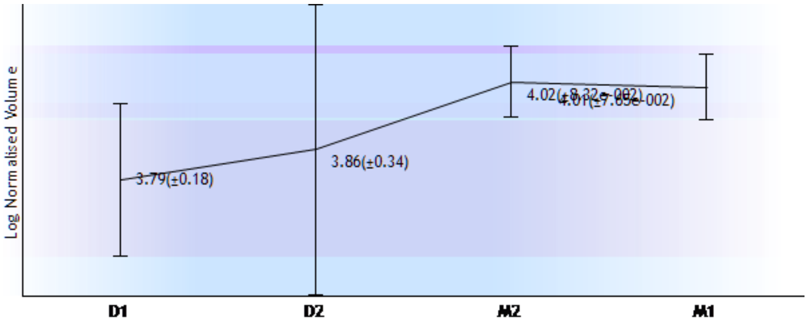

Identifier 595

Position (820, 455)

Notes

☐ Edited

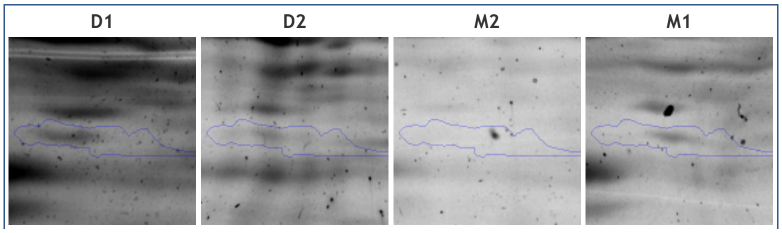

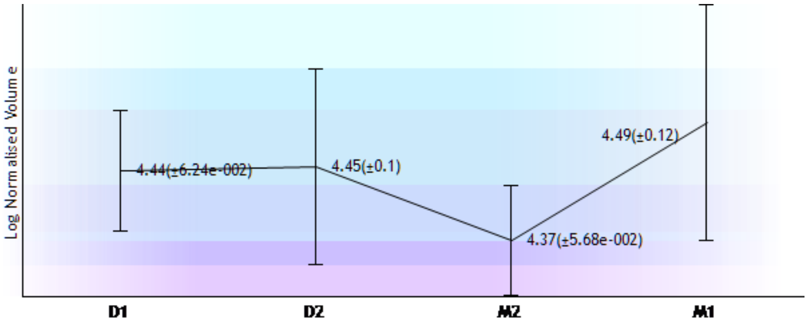

Identifier 431

Position (1108, 304)

Notes

☐ Edited

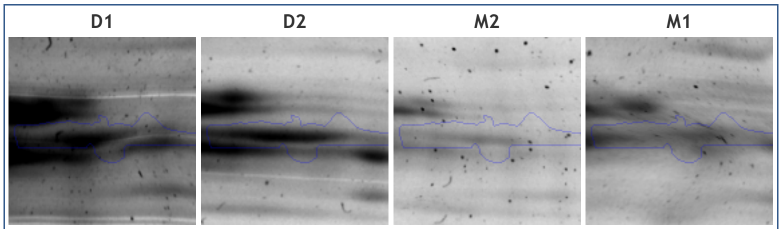

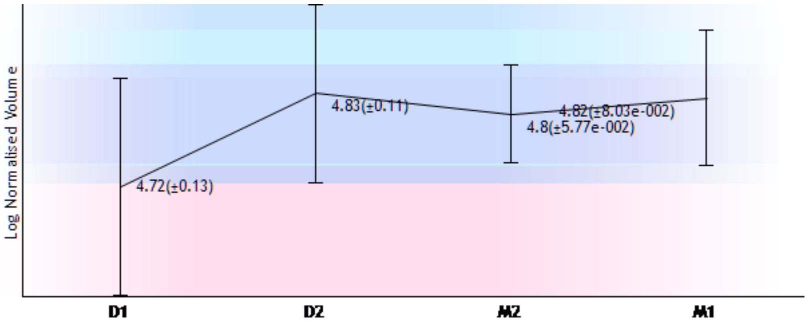

Identifier 596

Position (890, 502)

Notes

☐ Edited

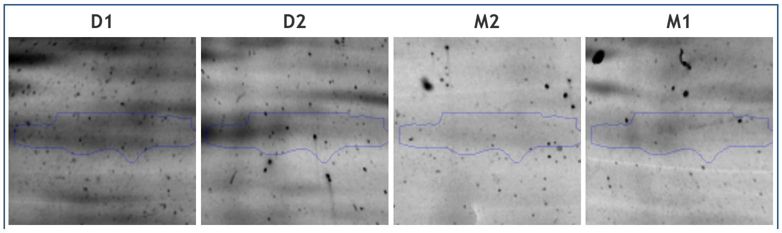

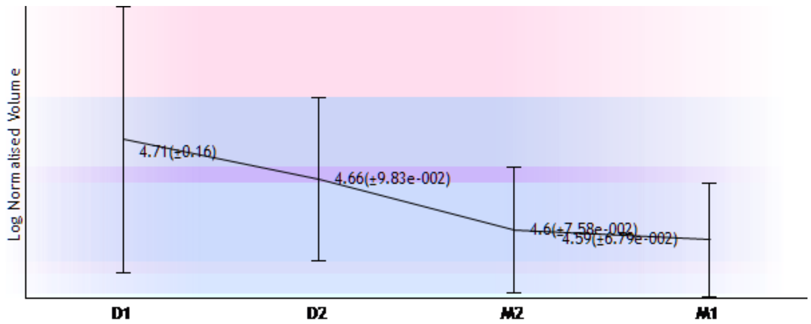

Identifier 337

Position (718, 581)
Notes

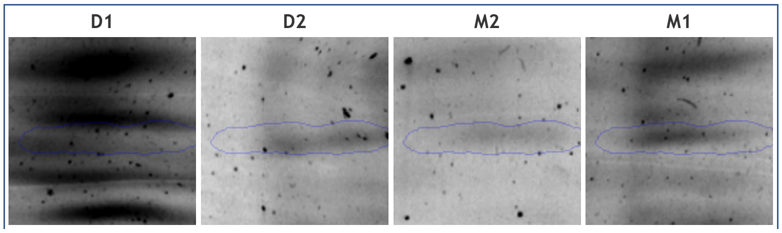

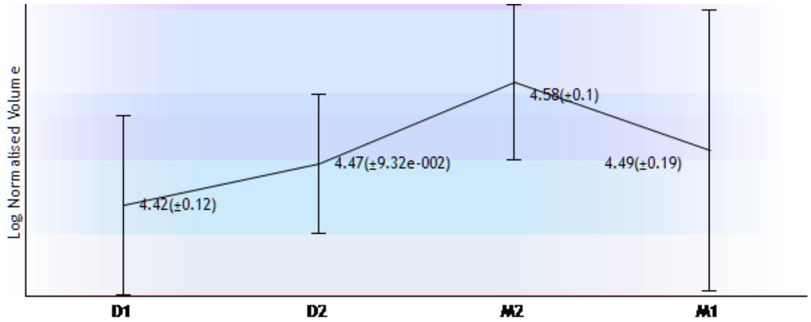

Identifier 611

Position (747, 308)
Notes
☐ Edited

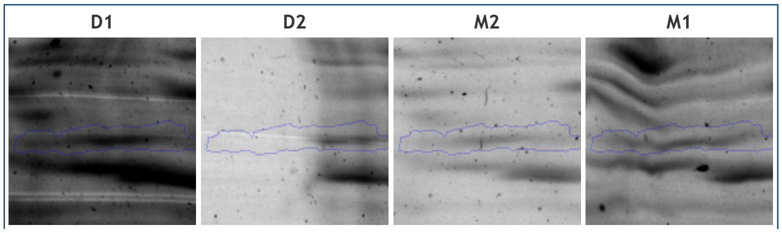

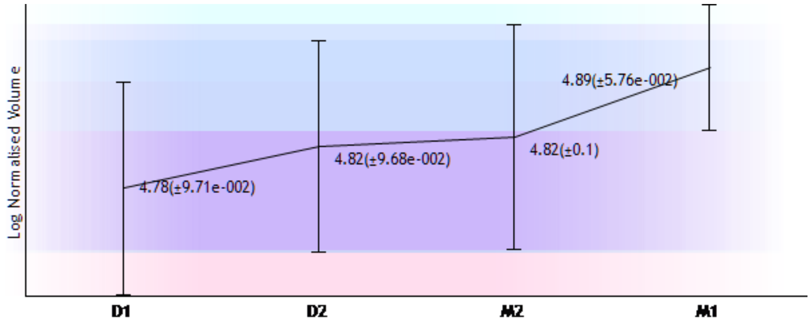

Identifier 529

Position (1109, 141)

Notes

Edited

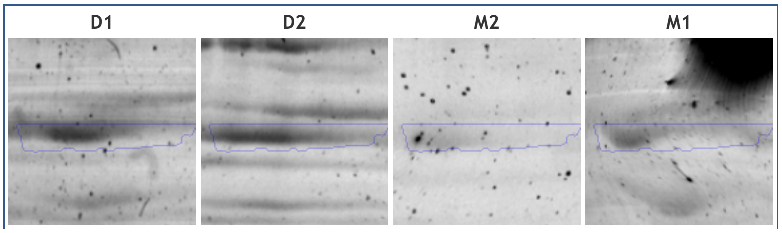

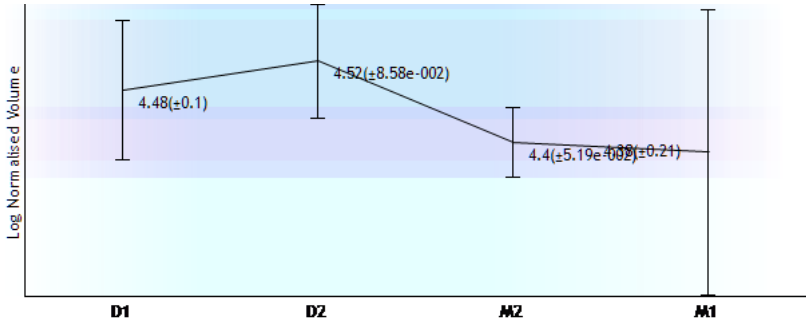

Identifier 541

Position (1170, 331)

Notes

Edited

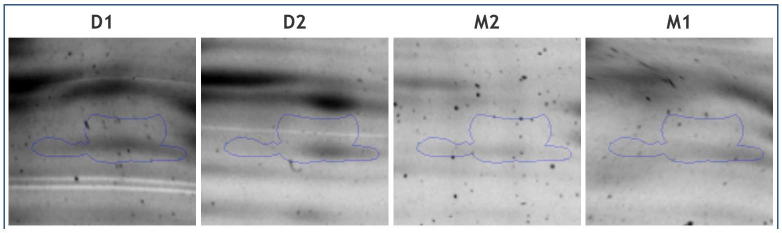

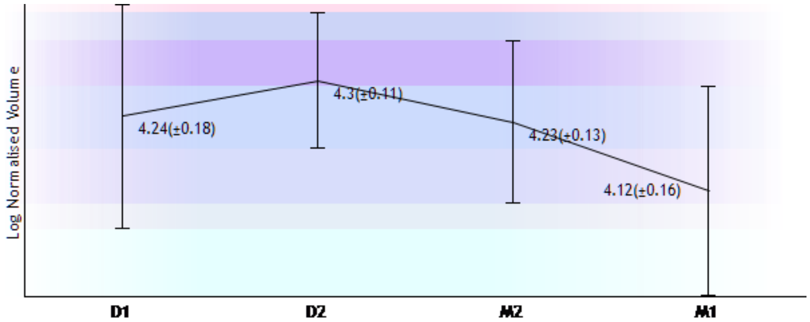

Identifier 645

Position (632, 333)

Notes

Edited

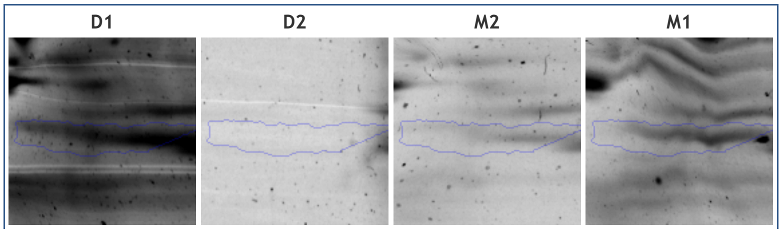

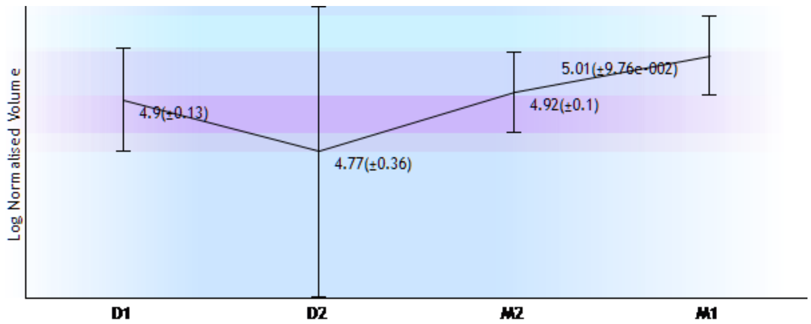

Identifier 494

Position (1023, 277)
Notes
☐ Edited

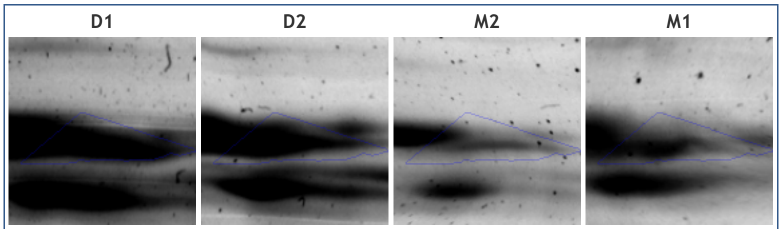

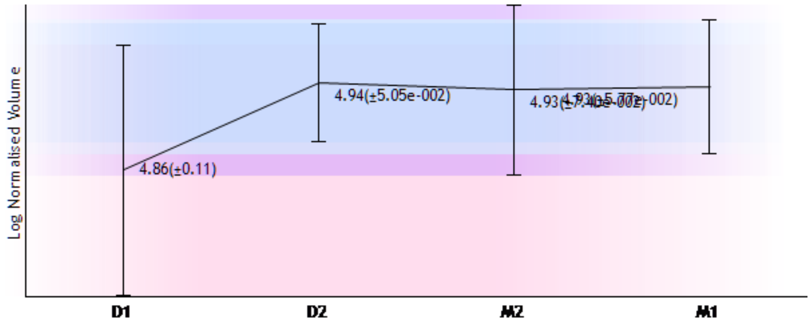

Identifier 589

Position (525, 376)
Notes
☐ Edited

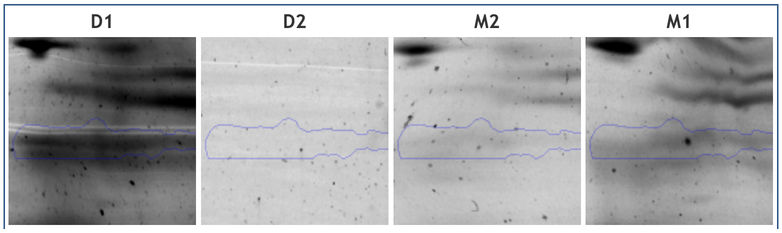

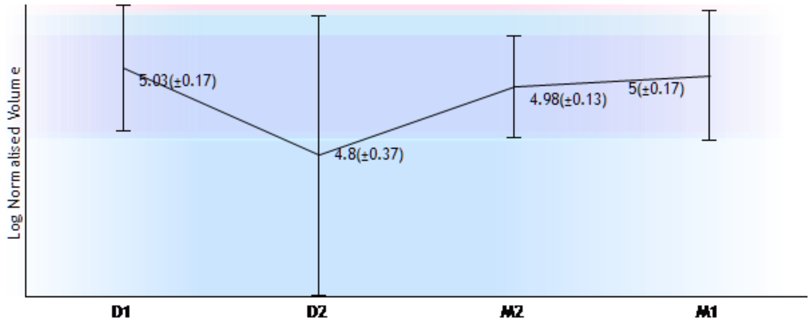

Identifier 492

Position (973, 271)

Notes

☐ Edited

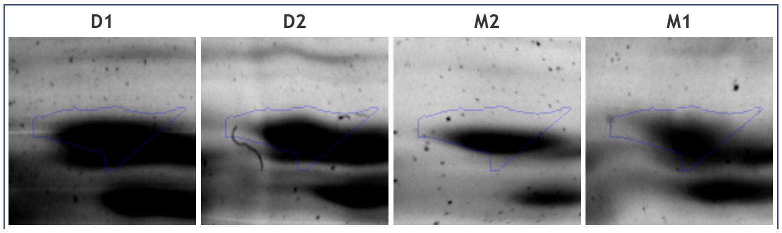

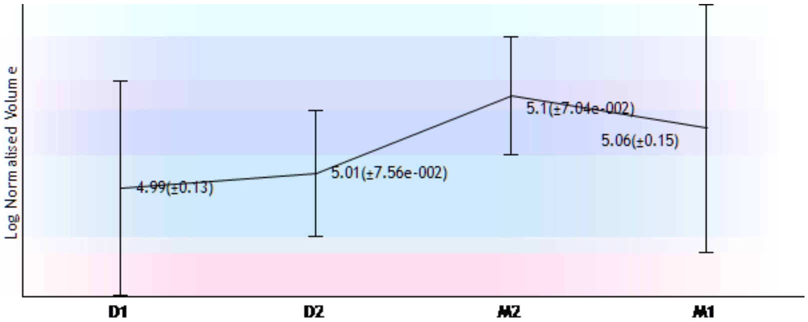

Identifier 501

Position (967, 277)

Notes

☐ Edited

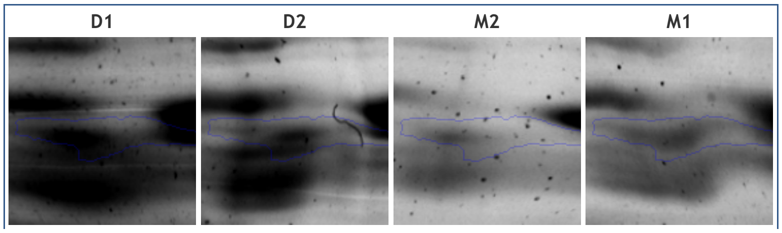

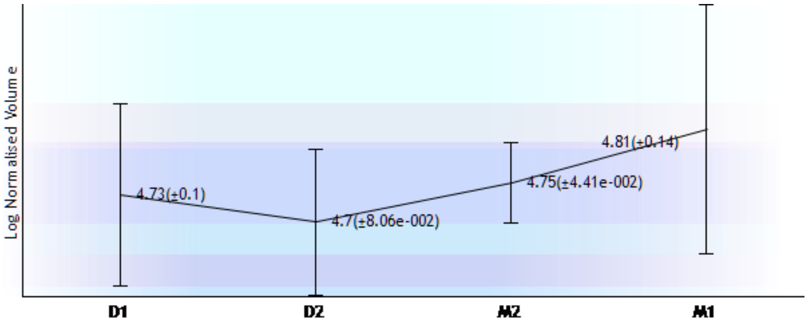

Identifier 605

Position (572, 614)

Notes

☐ Edited

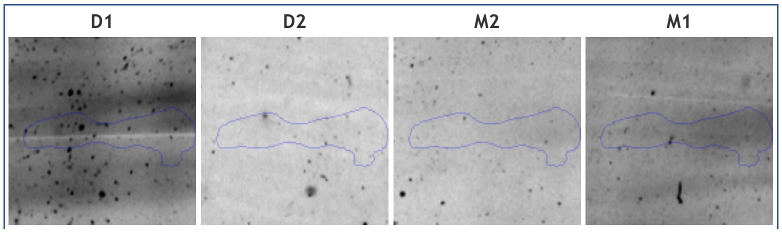

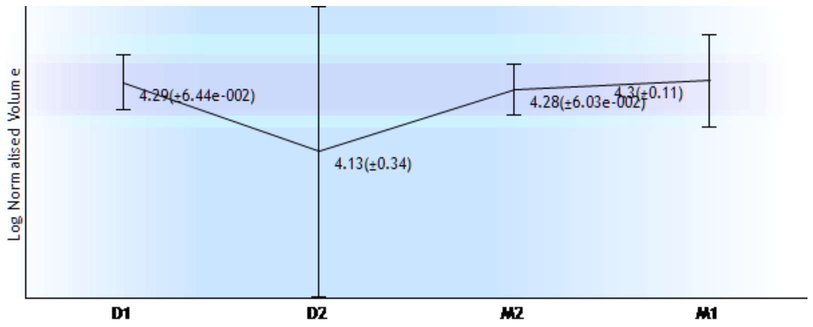

Identifier 270

Position (938, 440)
Notes

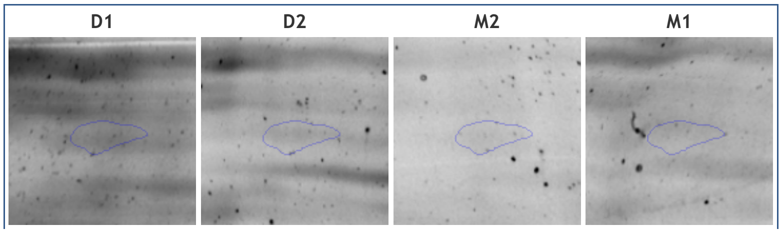

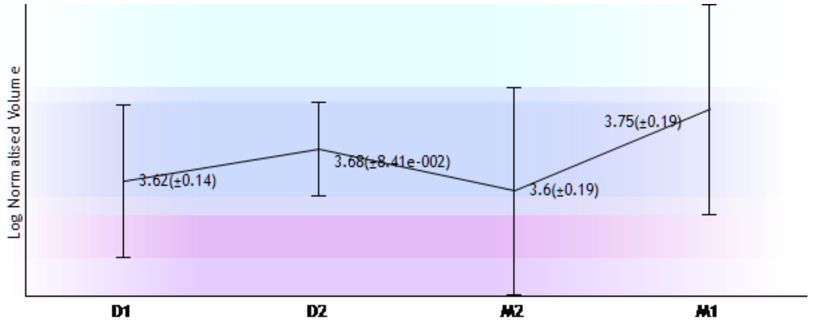

Identifier 603

Position (925, 391)
Notes
☐ Edited

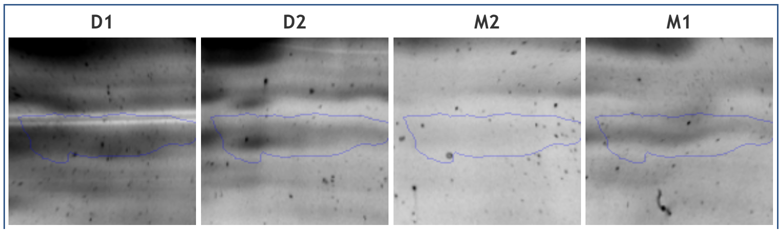

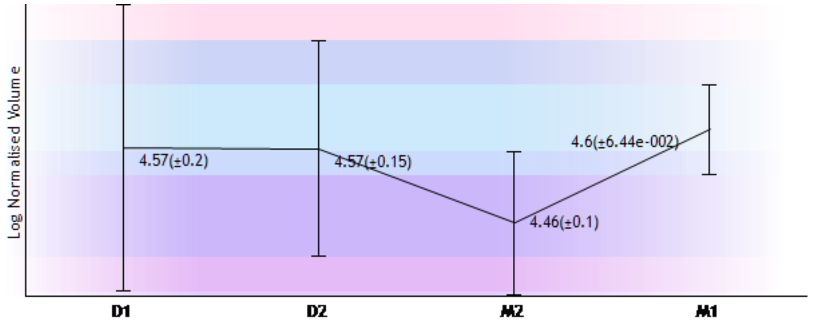

Identifier 578

Position (562, 505)

Notes

☐ Edited

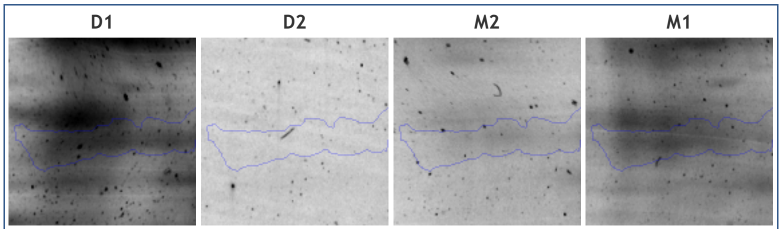

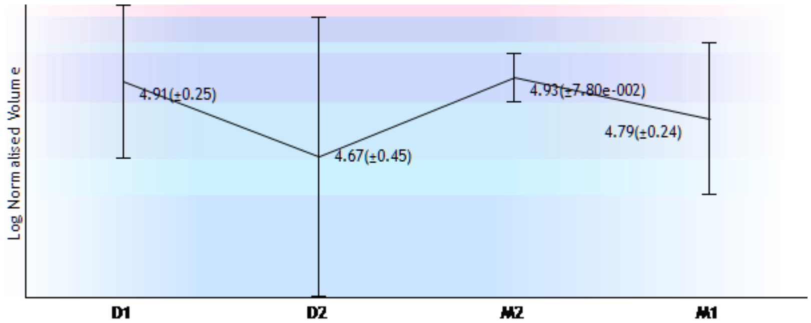

Identifier 647

Position (1032, 47)

Notes

☐ Edited

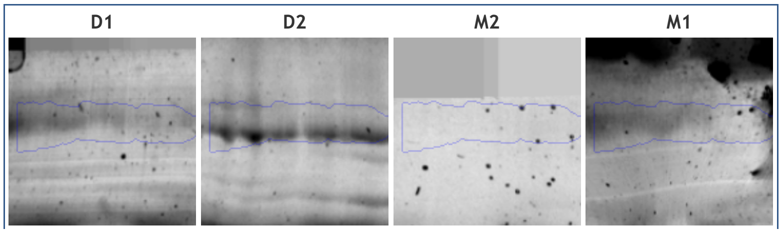

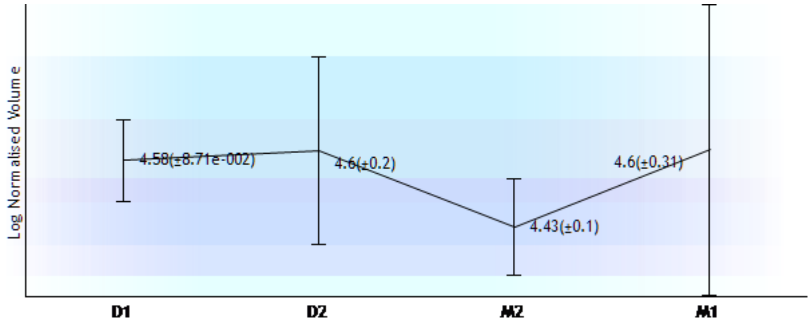

Identifier 471

Position (1052, 211)

Notes

☐ Edited

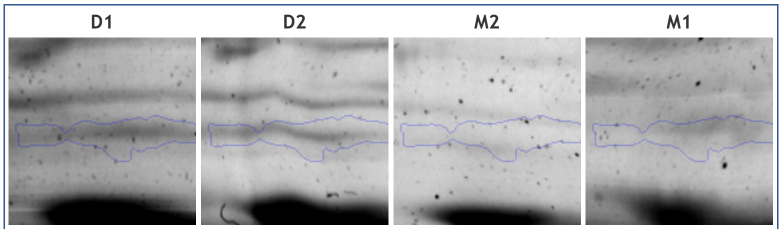

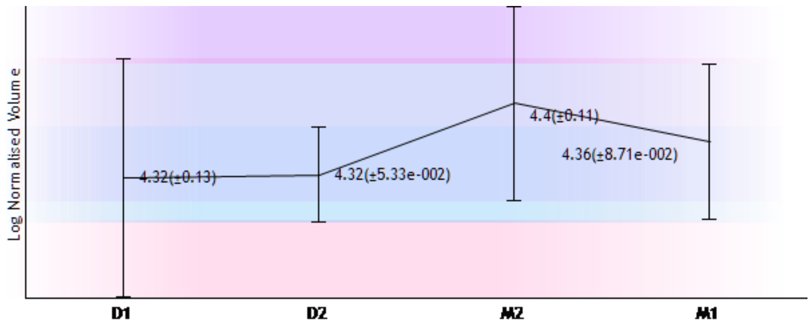

Identifier 484

Position (807, 262)

Notes

☐ Edited

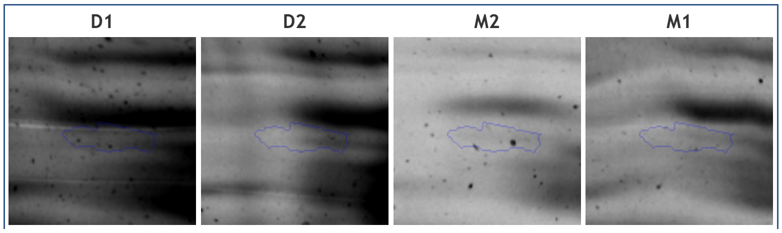

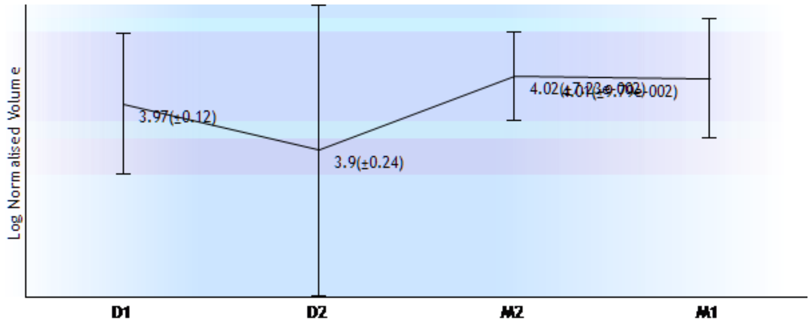

Identifier 9

Position (974, 55)

Notes

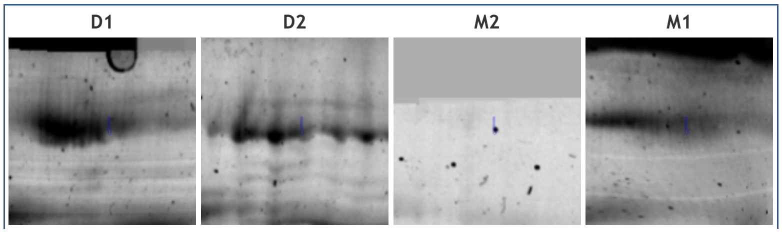

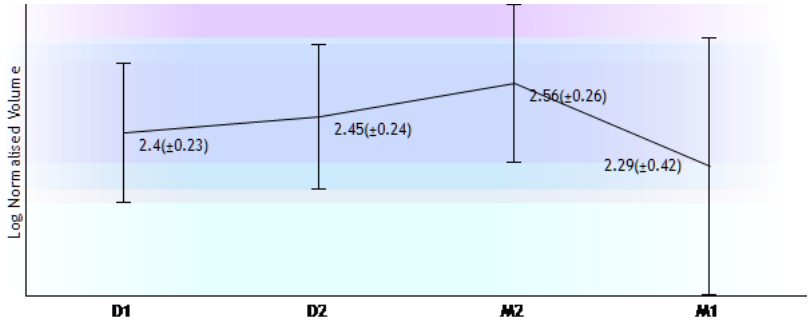

Identifier 440

Position (929, 355)

Notes

☐ Edited

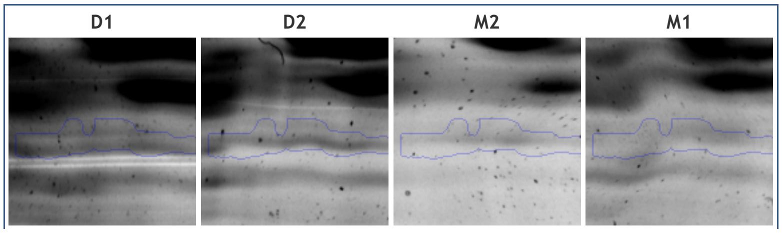

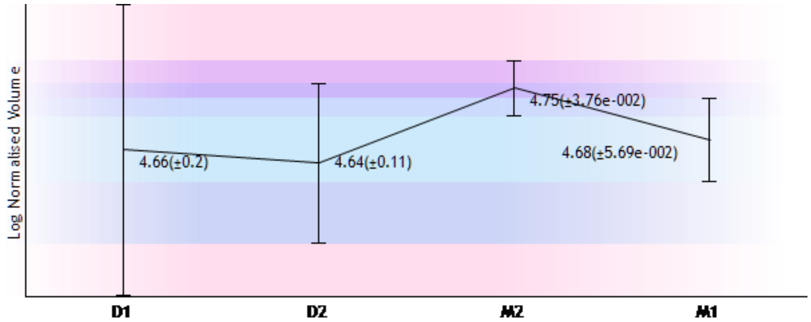

Identifier 636

Position (868, 197)

Notes

☐ Edited

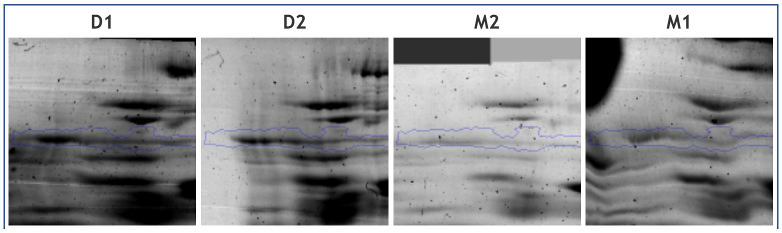

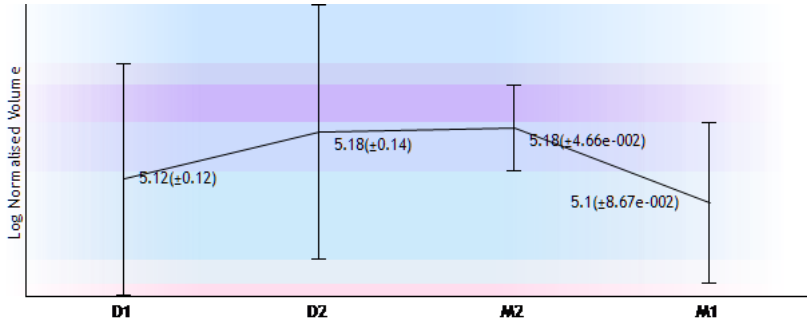

Identifier 587

Position (589, 410)

Notes

☐ Edited

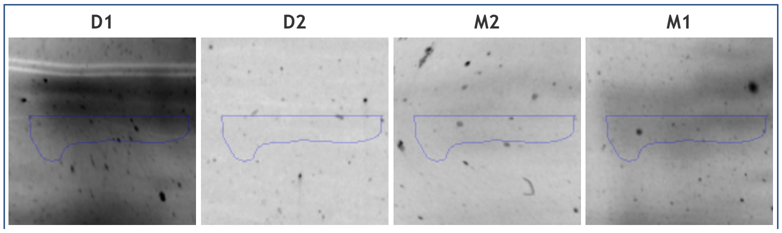

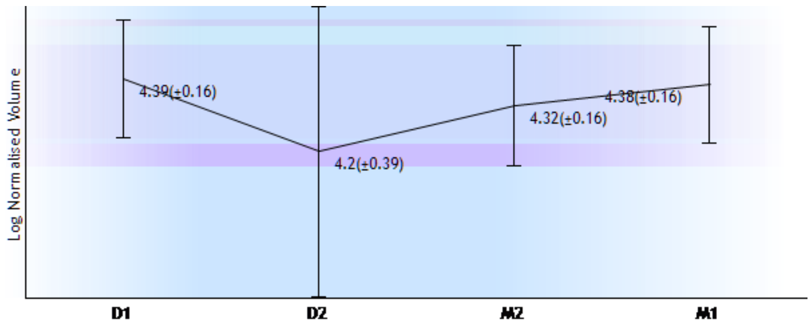

Identifier 451

Position (633, 282)
Notes
Edited

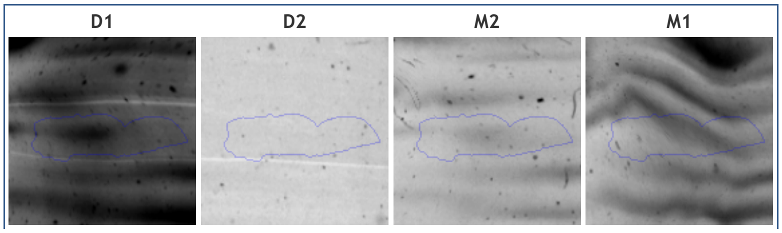

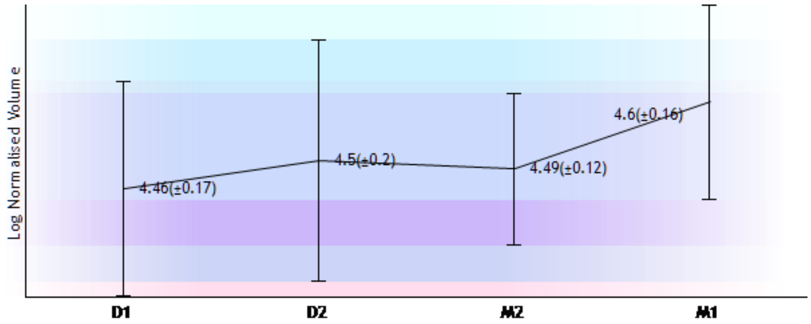

Identifier 512

Position (740, 634)
Notes
Edited

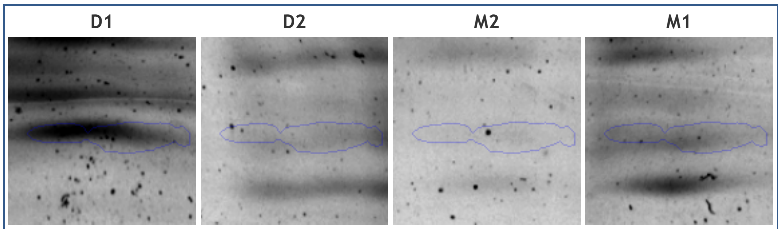

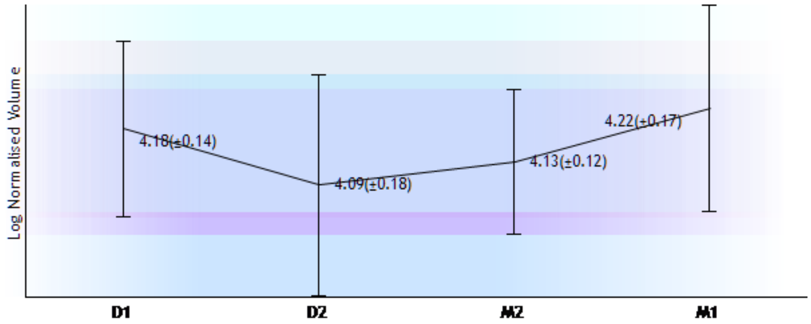

Identifier 618

Position (1099, 171)

Notes

☐ Edited

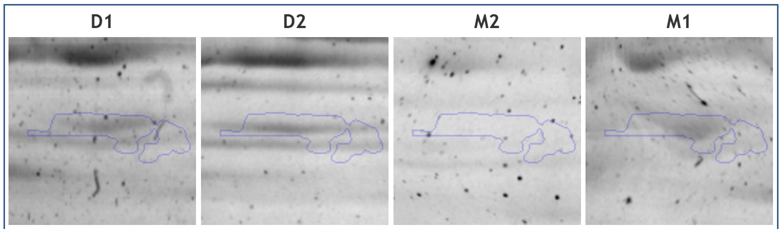

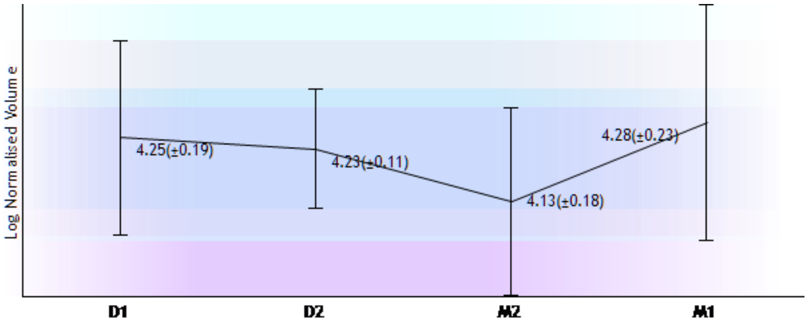

Identifier 626

Position (948, 152)

Notes

☐ Edited

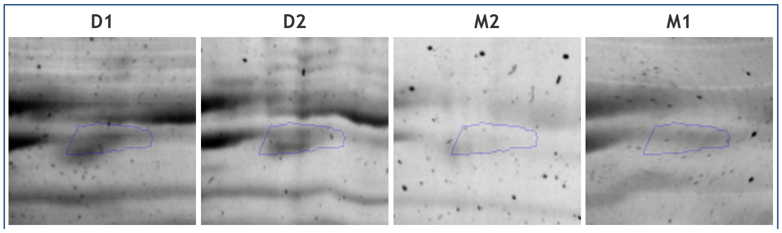

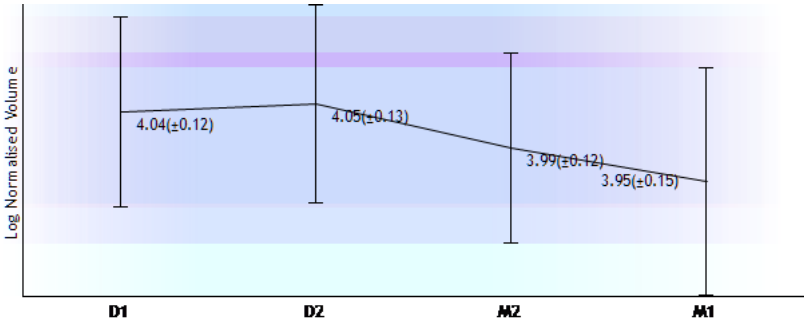

Identifier 530

Position (1099, 117)

Notes

☐ Edited

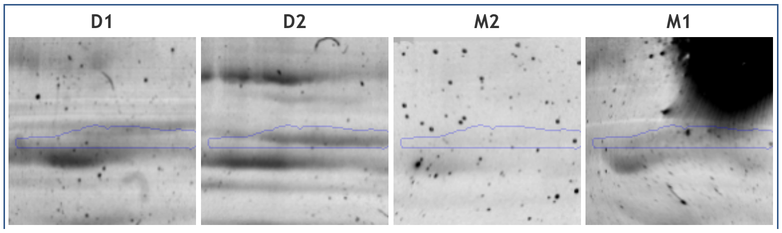

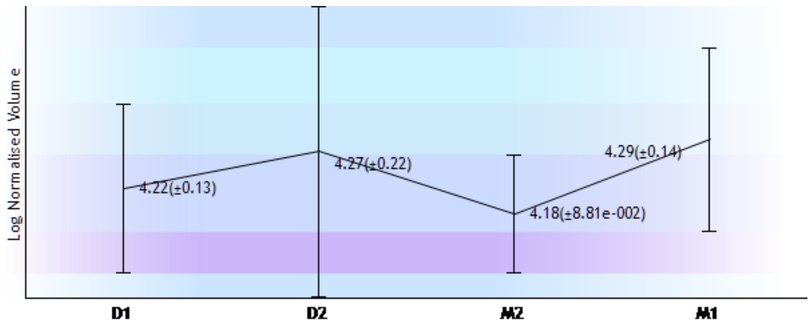

Identifier 592

Position (725, 543)
Notes
Edited

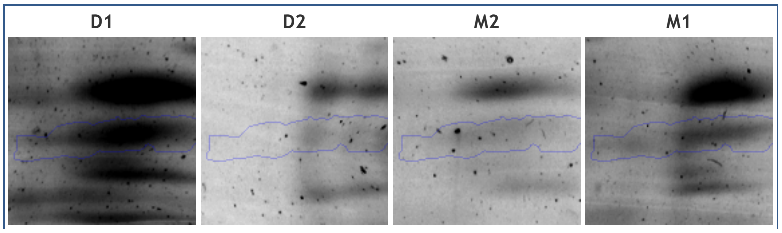

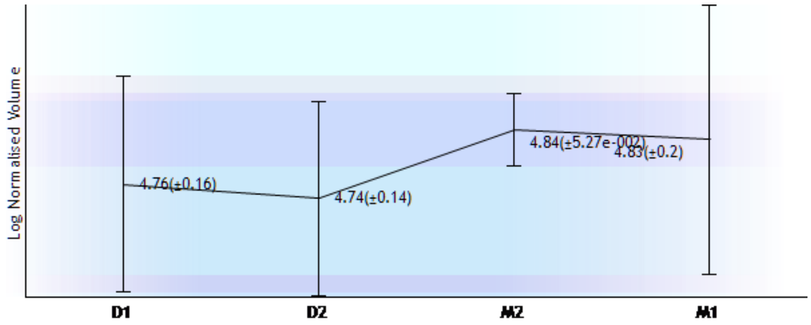

Identifier 570

Position (765, 678)
Notes
Edited

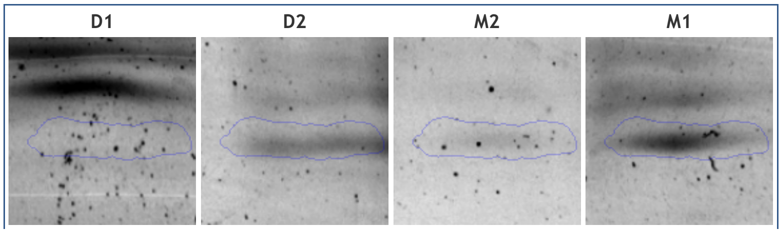

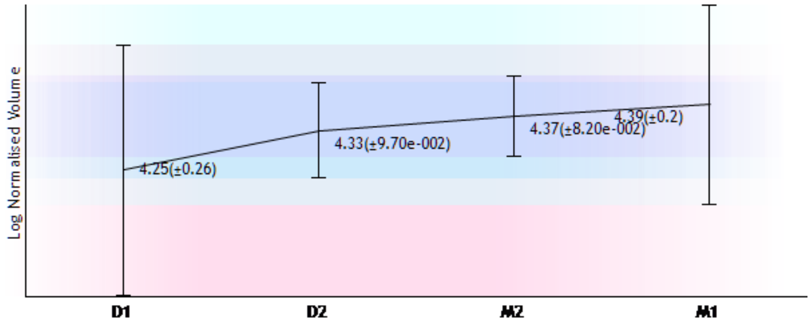

Identifier 324

Position (1369, 511)
Notes

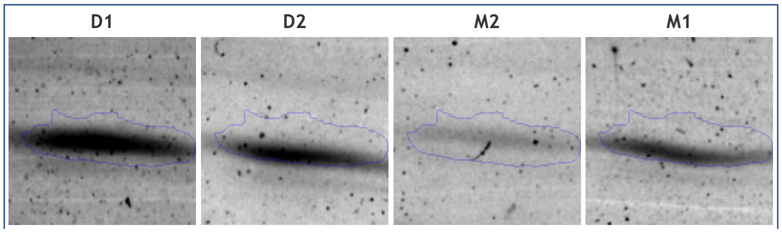

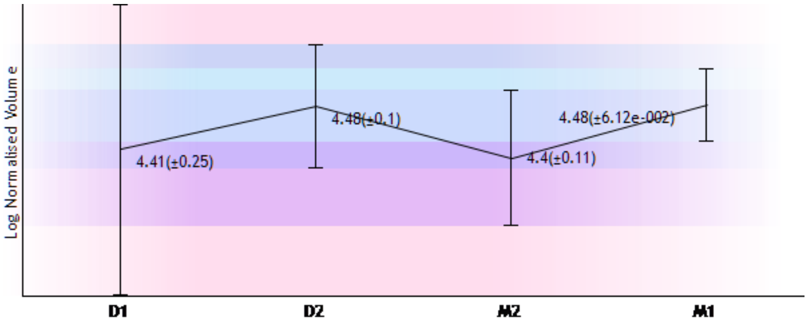

Identifier 585

Position (847, 122)
Notes
Edited

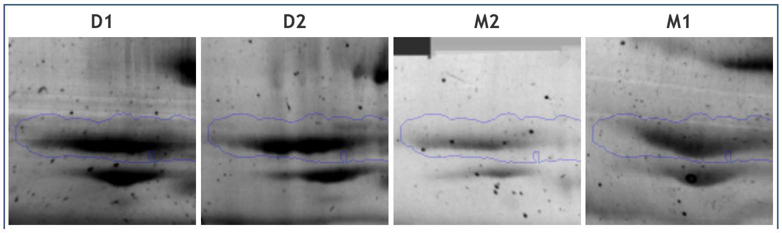

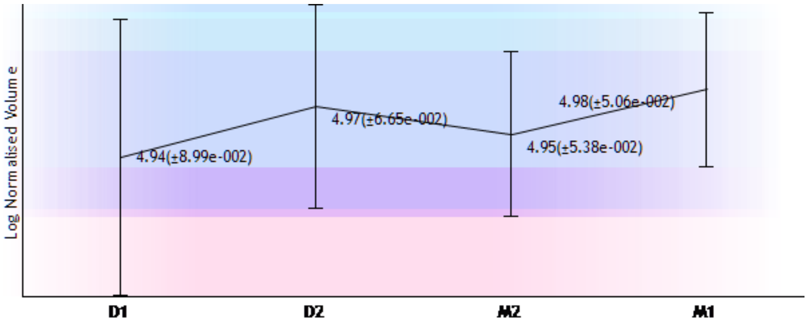

Identifier 651

Position (794, 377)
Notes
Edited

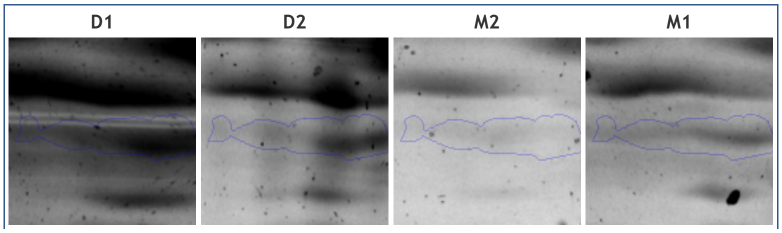

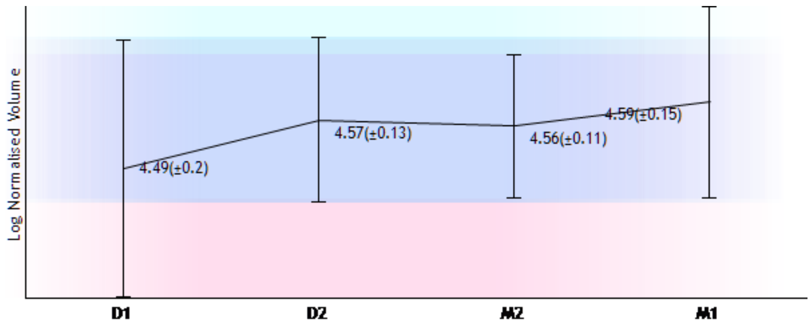

Identifier 106

Position (1167, 189)
Notes

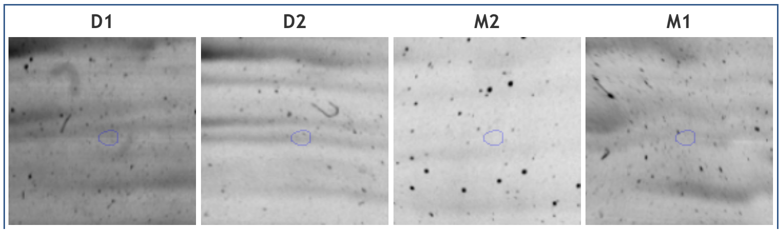

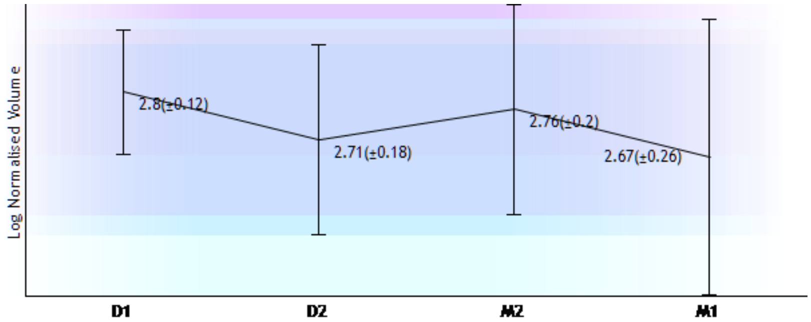

Identifier 532

Position (999, 134)
Notes
Edited

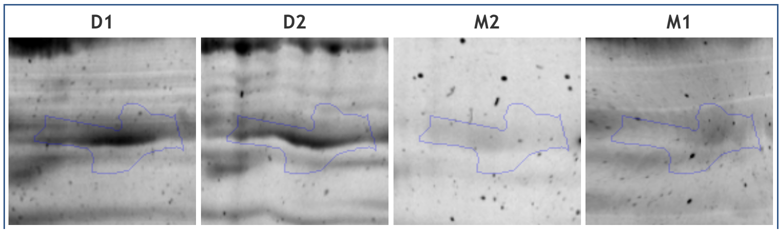

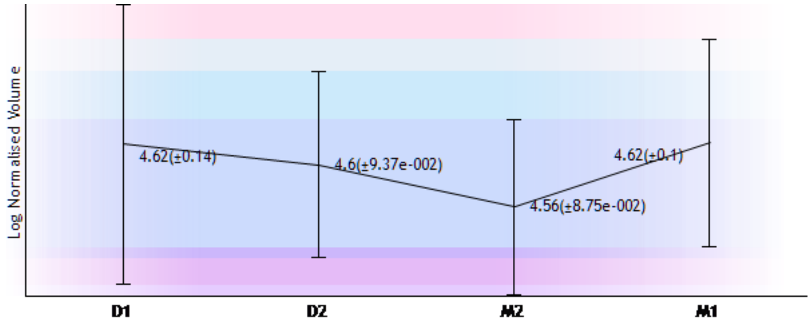

Identifier 644

Position (1046, 462)

Notes

☐ Edited

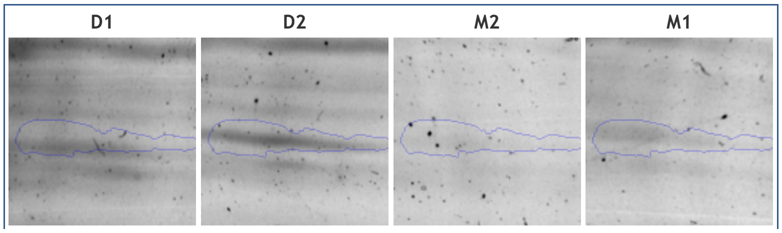

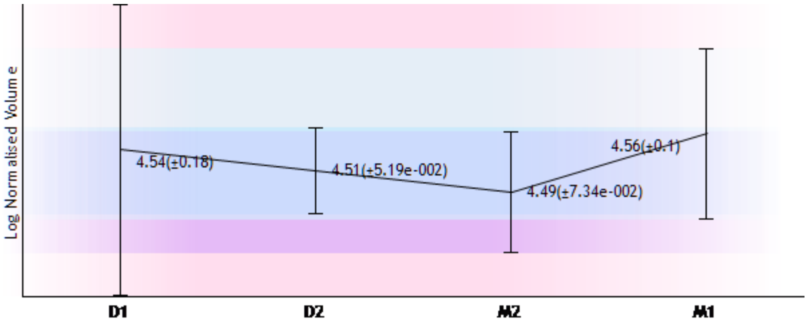

Identifier 648

Position (858, 259)

Notes

☐ Edited

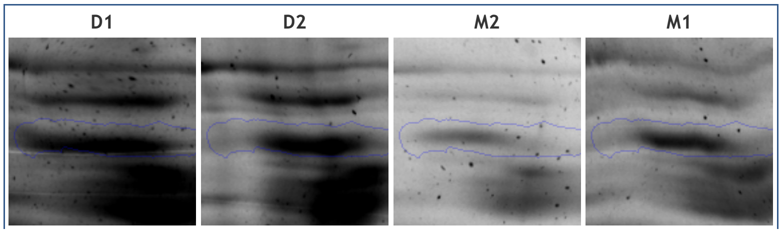

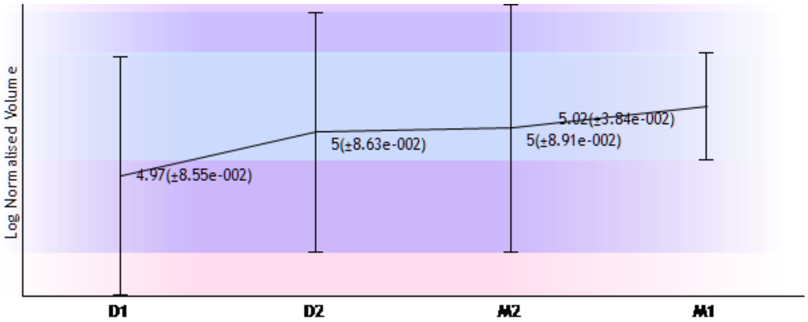

Identifier 602

Position (778, 350)

Notes

☐ Edited

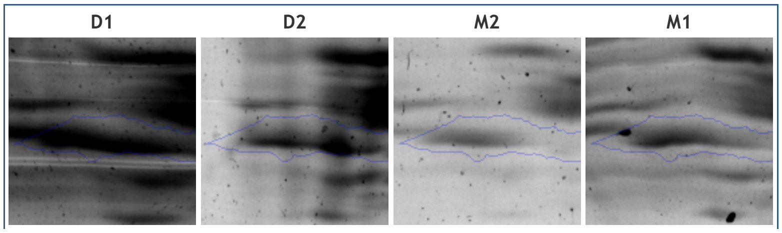

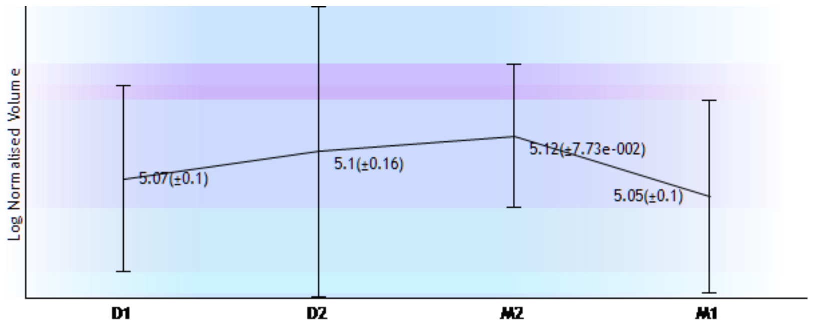

Identifier 620

Position (1160, 228)

Notes

Edited

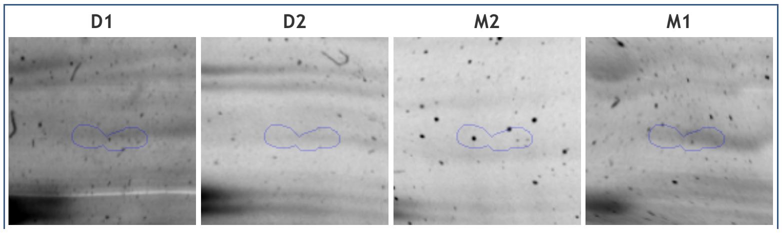

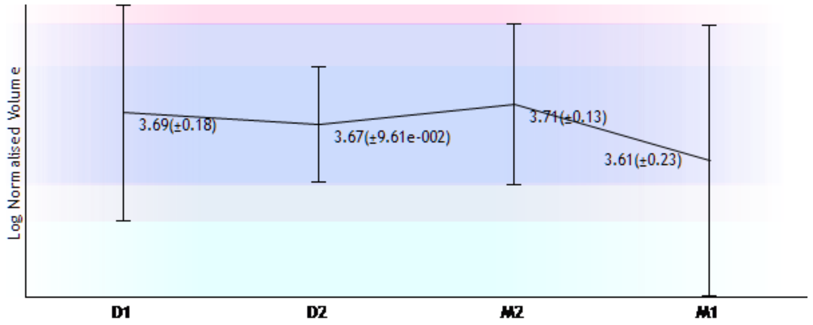

Identifier 628

Position (983, 312)

Notes

Edited

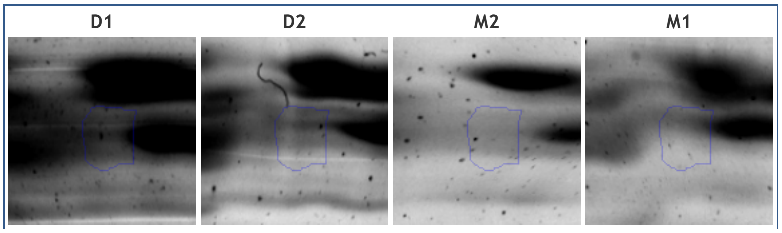

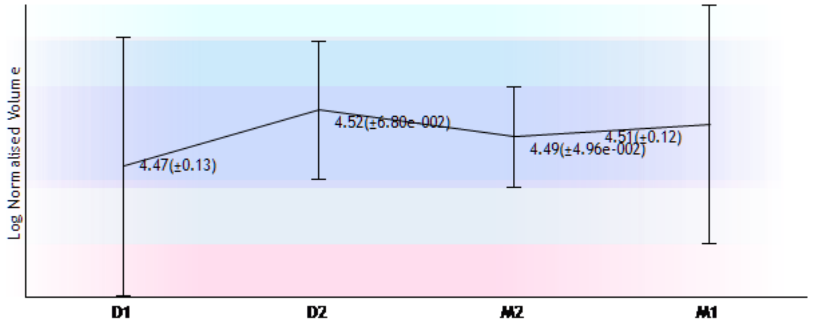

Identifier 625

Position (873, 140)

Notes

☐ Edited

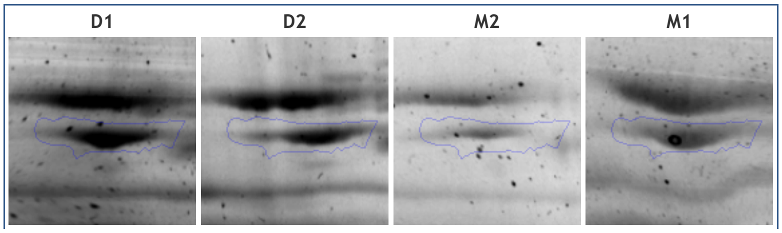

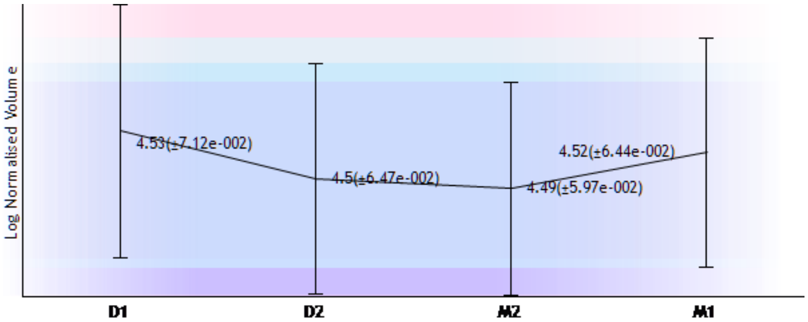

Identifier 183

Position (1334, 292)

Notes

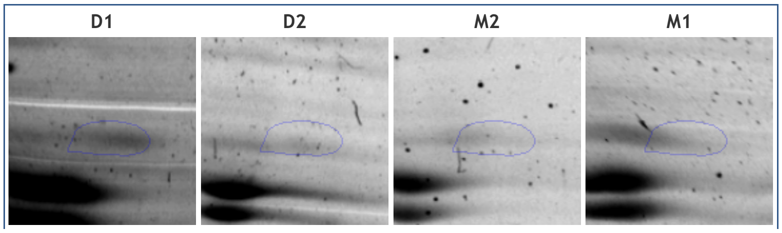

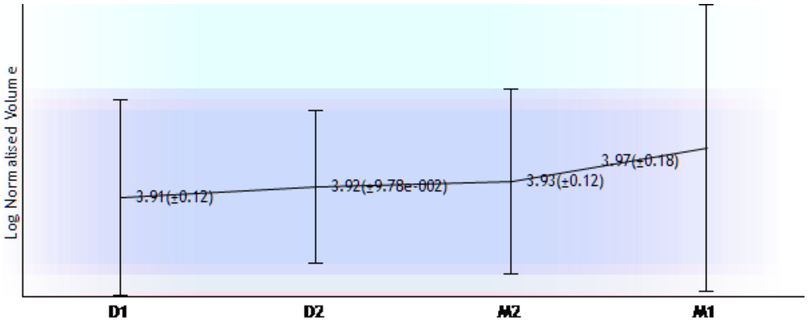

Identifier 619

Position (1092, 186)

Notes

☐ Edited

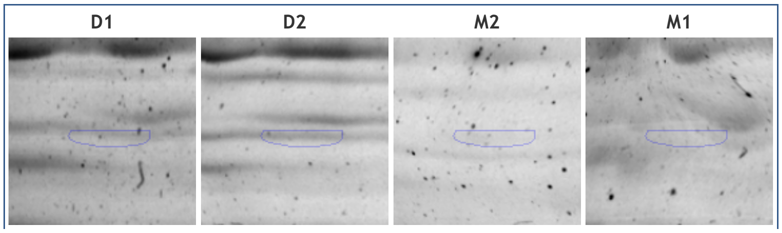

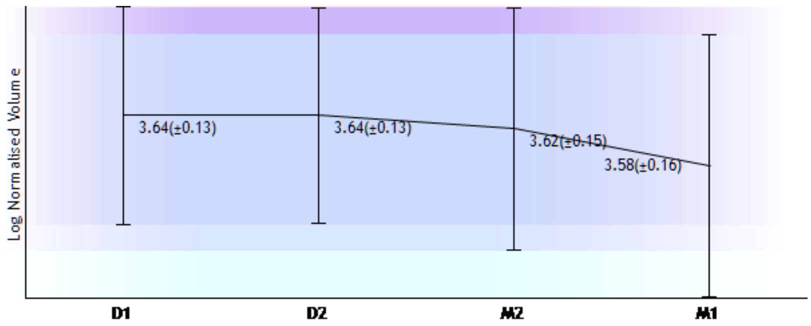

Identifier 355

Position (854, 636)
Notes

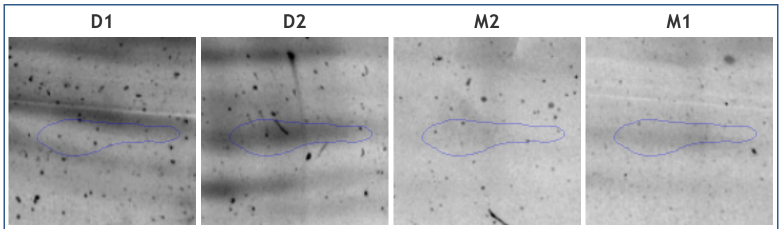

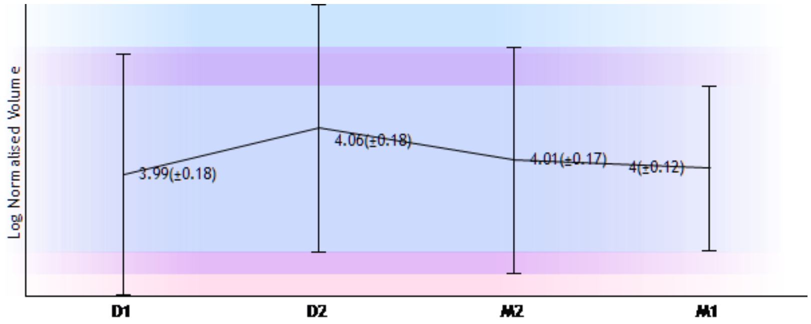

Identifier 638

Position (620, 185)
Notes
Edited

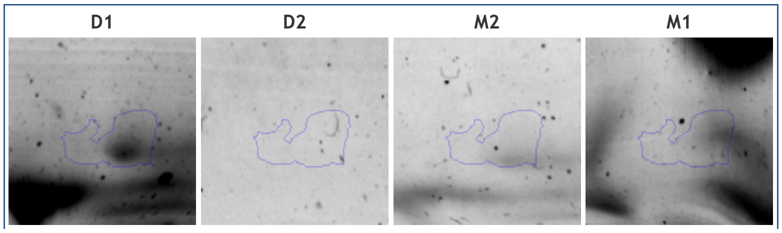

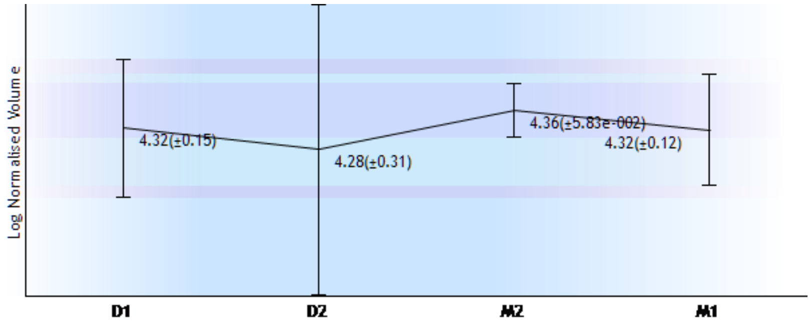

Identifier 630

Position (1051, 390)

Notes

☐ Edited

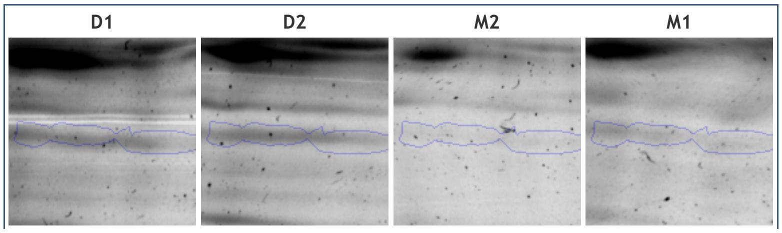

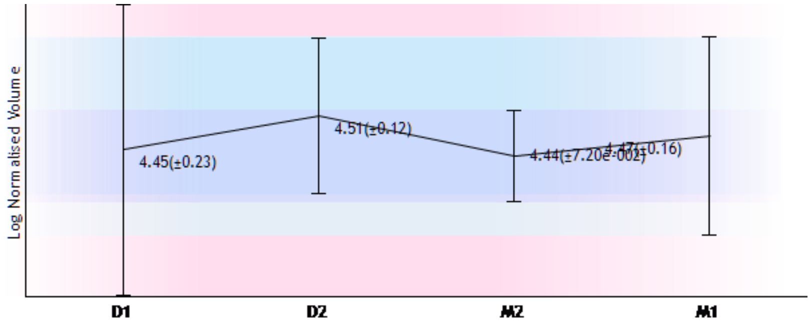

Identifier 567

Position (817, 435)

Notes

☐ Edited

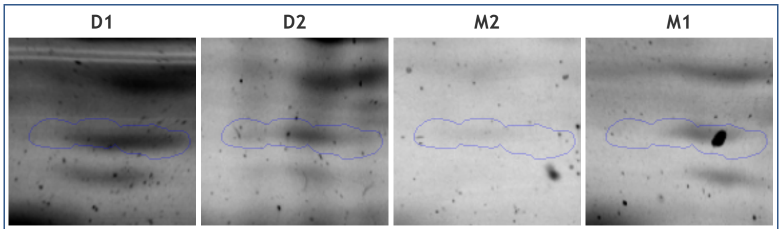

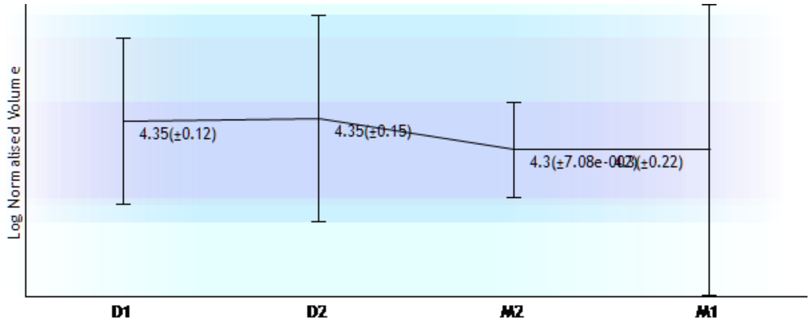

Identifier 486

Position (879, 218)

Notes

☐ Edited

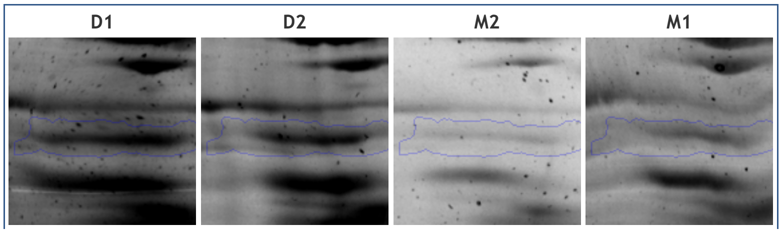

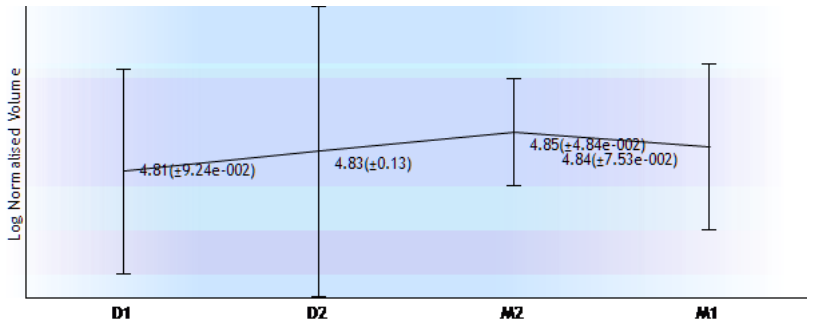

Identifier 632

Position (1088, 271)

Notes

Edited

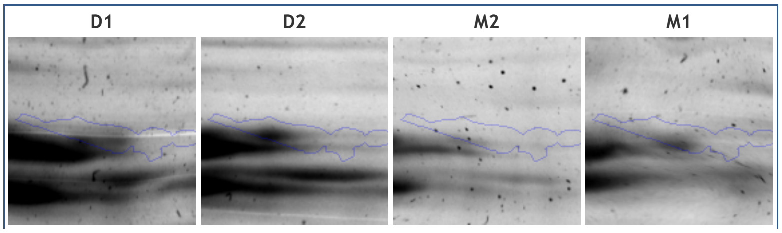

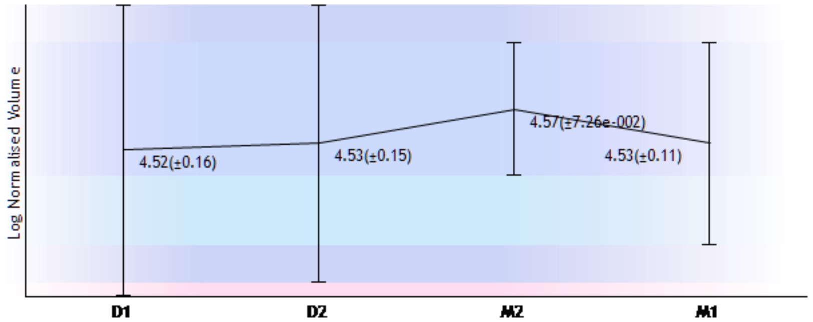

Identifier 609

Position (1456, 335)

Notes

Edited

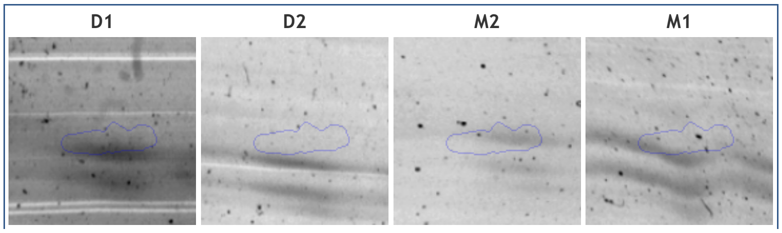

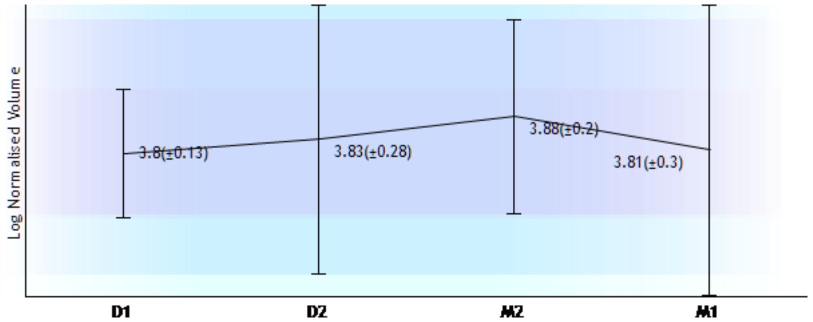

Identifier 643

Position (687, 251)

Notes

☐ Edited

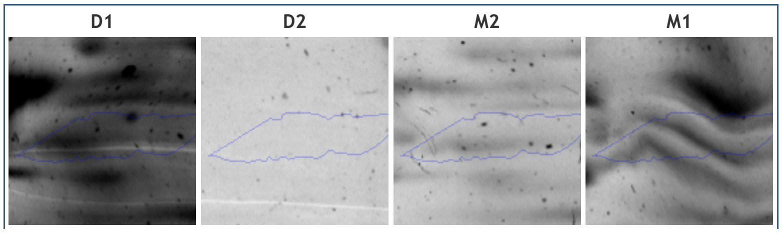

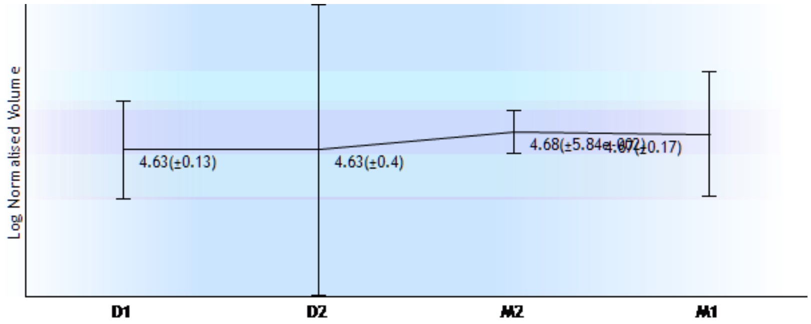

Supplement: S1 Report — (PDF) [file pone.0212580.s001.pdf]
